# Characterization of *Pseudomonas aeruginosa* and *Acinetobacter calcoaceticus-baumannii* complex traumatic wound isolates

**DOI:** 10.1128/spectrum.02644-25

**Published:** 2026-06-12

**Authors:** Ian H. Windham, Jeffrey A. Kimbrel, Olivia A. Boykin, Marina R. Wylie, Mark P. Simons, Seth A. Schobel, Michael Rouse, Eric A. Elster, Christopher J. Dente, Nicholas A. Be, Joshua Stanbro, Jennifer Huber, Meenu M. Upadhyay, Angela R. Melton-Celsa, D. Scott Merrell

**Affiliations:** 1 Department of Microbiology and Immunology, Uniformed Services University of the Health Sciences, Bethesda, Maryland, USA; 2 Henry M. Jackson Foundation for the Advancement of Military Medicine, Inc., Bethesda, Maryland, USA; 3 Physical and Life Sciences Directorate, Lawrence Livermore National Laboratory, Livermore, California, USA; 4 Naval Medical Research Command, Silver Spring, Maryland, USA; 5 Surgical Critical Care Initiative (SC2i), Uniformed Services University of the Health Sciences (USUHS), Bethesda, Maryland, USA; 6 Department of Surgery and Grady Memorial Hospital, Emory University School of Medicine 12239, Atlanta, Georgia, USA; 7 Animal and Comparative Biomedical Sciences, University of Arizona, Tucson, Arizona, USA; College of New Jersey, Ewing, New Jersey, USA

**Keywords:** *Pseudomonas aeruginosa*, *Acinetobacter*, biofilms, traumatic wound

## Abstract

Healing of traumatic wounds is complicated by infecting pathogens, with *Pseudomonas aeruginosa* and members of the *Acinetobacter calcoaceticus-baumannii* complex among the most common infectious agents. However, a full understanding of genotypic and phenotypic differences between circulating wound isolates is lacking. To address this, traumatic wounds were sampled at Emory University Hospital, and 24 isolates were characterized; we focused on *P. aeruginosa* and *Acinetobacter* due to their prevalence and tendency for antibiotic resistance. Even though these species are renowned for antibiotic resistance, only two of the tested isolates could be classified as multidrug resistant. Whole-genome sequencing and analysis revealed that isolates from different patients were genetically distinct; however, longitudinal isolates from the same patient were closely related and appeared to represent chronic colonization by the same strain. Phylogenetic analysis revealed that laboratory strains (PAO1 and AB5075) that were isolated decades previously and from different locations grouped closely to subsets of the recent wound isolates. Given the importance of biofilm formation in infection, the ability of the isolates to form biofilms was assessed; all isolates formed biofilms but showed temporal and magnitude differences. Analysis of a subset of isolates revealed that planktonic *P. aeruginosa* was resistant to human serum-mediated killing. While the same was true for the majority of *Acinetobacter* isolates, one showed planktonic sensitivity that was abrogated when grown in a biofilm. Taken together, these data reveal genotypic and phenotypic differences in circulating isolates of *P. aeruginosa* and *A. baumannii*.

**IMPORTANCE:**

The presence or absence of bacterial virulence factors and antibiotic resistance genes can greatly alter disease progression in a traumatic wound as well as the best course of treatment. Therefore, there is a need for a greater understanding of the genotypic and phenotypic differences between circulating wound isolates. Characterization of clinical isolates of *P. aeruginosa* and the *A. calcoaceticus-baumannii* complex obtained from traumatic wounds at Emory University Hospital revealed genotypic and phenotypic differences that resulted in variation in antibiotic resistance, biofilm formation, and the presence of potential virulence factors. A greater understanding of wound isolates may provide avenues for better therapies for the treatment of future infections. Taken together, these data reveal genotypic and phenotypic differences in circulating isolates of *P. aeruginosa* and *A. baumannii*.

## INTRODUCTION

With the Medicare cost of wound treatments alone estimated to be up to $97 billion in 2018 (1), traumatic wounds represent a major burden to the healthcare system, in part due to infection by environmental bacteria. These infections are associated with substantial morbidity, long-term complications, and subsequent increased burden on the wound care and healthcare systems (2); the cost of chronic infection treatment alone is estimated to be more than $25 billion annually in the United States (3). While many different species can be introduced into the wound during the trauma, prior studies indicate that gram-positive species are commonly identified at this stage (4–6). However, as time progresses, gram-negative species like *Pseudomonas aeruginosa* and *Acinetobacter baumannii* are often found to dominate the wound bed (7); this makes a greater understanding of wound-associated isolates of these two species of great importance (8, 9).

It is estimated that *P. aeruginosa* is responsible for 10%–15% of all nosocomial infections (10), while *A. baumannii* is responsible for 2%–10% of the hospital infections that are caused by gram-negative bacteria (11). *P. aeruginosa* is noted for the ability to cause disease in patients with cystic fibrosis, cancer, HIV, and severe burns (9, 12, 13), and *A. baumannii* is often isolated from skin and soft tissue infections from patients suffering from severe burns or wounds (14, 15). Treatment options for *P. aeruginosa* remain limited because antibiotic resistance is increasing (16); moreover, mortality rates remain high despite new antimicrobials that have been developed and employed (17). Similarly, treatment of *A. baumannii* infections is complicated/limited by the species’ ability to acquire a high level of multi-drug resistance (18). As such, antibiotic-resistant strains of both bacterial species are recognized as serious threats to human health by the World Health Organization (19) and the Centers for Disease Control and Prevention (20).

In addition to the highlighted challenge of antibiotic resistance, biofilm formation is also known to affect treatment outcomes. Biofilms are a collection of surface-adhered cells that are encased by an extracellular matrix composed of assorted proteins, polysaccharides, and extracellular DNA (21) that are generated by the bacteria (22). Existence within the biofilm creates a heterogeneous environment (23), which increases the ability of the bacteria to tolerate antibiotic treatment (24, 25) and protects the bacterial cells from the immune system (26–29). Biofilms are an important component of the lifecycle of many infections; indeed, it is estimated that more than 90% of bacterial infections feature biofilms as a mode of growth (19). Both *P. aeruginosa* and *A. baumannii* are well known to establish biofilms (30, 31).

Given that environmental contamination can seed bacteria directly into the wound, analysis of clinical isolates from wounds can provide a snapshot of the bacterial pathogens that are found within the local environment that are able to successfully establish infection (32, 33). Moreover, genotypic and phenotypic analysis of these clinical isolates can be used as a means to define patient or even geographic differences in circulating pathogenic species. Additionally, given the extended length of time that it takes for some traumatic wounds to heal, longitudinal sampling and analysis of isolates from the same wound can reveal important information about the dynamics of the infection process as well as microevolution within a species as individual isolates within an infection can change over time and can also adapt to the conditions of the host (34).

To expand our understanding of genotypic and phenotypic differences between circulating wound isolates of *P. aeruginosa* and *Acinetobacter*, we analyzed 24 isolates obtained from traumatic wounds at Emory University Hospital. Single isolates collected from individual patients and longitudinal isolates obtained from the same patients were included. Despite the well-known tendency of these species to exhibit high rates of antibiotic resistance, only two of the *P. aeruginosa* isolates were multidrug resistant. Whole genomic sequence and phylogenetic analysis showed that isolates from the same patient were often nearly identical and distinct from isolates obtained from different patients. Furthermore, common laboratory strains that were isolated decades ago (PAO1 and AB5075) were genetically similar to study isolates of this study within their respective species. We found that all of the isolates formed biofilms but showed temporal and magnitude differences depending on the specific biofilm assay. Moreover, planktonic *P. aeruginosa* isolates were resistant to human serum-mediated killing, as were the majority of *Acinetobacter* isolates; the one *Acinetobacter* isolate that showed planktonic serum sensitivity became serum resistant when grown in a biofilm. Taken together, these data reveal genotypic and phenotypic differences in circulating strains of *P. aeruginosa* and *A. baumannii*.

## MATERIALS AND METHODS

### Bacterial isolates and growth

The strains and isolates used in this study are listed in Table 2. Isolates were collected as part of the Uniformed Services University Surgical Critical Care Initiative Emory-WounDx study (IRB #58229) at Grady Hospital/Emory University Hospital in Atlanta, Georgia, as described previously (35, 36). Briefly, wound effluent (~20–30 mL) from negative pressure wound therapy was collected in the operating room before each surgical debridement. The samples were then centrifuged at 2,500 g at 4°C for 10 min, and supernatant aliquots were transferred to labeled Cryo-Loc polypropylene tubes (Lake Charles Manufacturing, Lake Charles, LA, USA). Aliquots were flash-frozen in liquid nitrogen and stored at −80°C for batch analysis.

Individual isolates were collected and characterized as follows: for effluent (liquid), 10 μL of the effluent was pipetted into 10 mL of Fastidious Broth to achieve a 10^6^ dilution. After mixing, 1 μL of the Fastidious Broth/effluent mixture was placed onto sheep’s blood agar plates and spread utilizing a sterile loop; a 10^5^ dilution was achieved by pipetting a 10 μL aliquot. For tissue (solid) samples, the samples were homogenized in 1 mL of Fastidious Broth using a Kendall disposable tissue grinder. Additional Fastidious Broth (nine times tissue weight) was added to each tissue sample based on its weight (e.g., 0.2 g tissue required 1.8 mL total volume). For the 10^5^ plate, 10 μL of the homogenate was added onto a sheep’s blood agar plate in duplicate and spread using a sterile loop. For the 10^6^ plate, 1 μL of the homogenate was added onto a sheep’s blood agar plate in duplicate and spread using a sterile loop. Agar plates were incubated overnight at 37°C. Bacterial colonies were enumerated to obtain an accurate and pure colony count and expressed as either CFU per milliliter of effluent or CFU per gram of tissue. Individual colonies were selected and identified as gram-positive or gram-negative via standard Gram stain according to the instructions of the manufacturer (Sigma-Aldrich). Further identification and antibiotic sensitivity were determined using the BD Phoenix automated identification and susceptibility system (Franklin Lakes, NJ, USA) following the manufacturer’s protocol and reagents (37, 38). Briefly, pure colonies from overnight culture plates were suspended in the Phoenix ID broth and vortexed to generate a uniform suspension to a density of 0.5 McFarland as determined using the BD Phoenix Spec nephelometer. Twenty-five microliters of the 0.5 McFarland suspension was added to the antibiotic susceptibility testing (AST) broth and mixed gently by inversion to avoid generating bubbles. The ID and AST broths were added by pouring into the respective inoculation port of the PMIC/ID-107 panel (number 448607). Panels were placed into the instrument and run to completion (18–24 h). Inocula were plated on tryptic soy agar overnight to further ensure culture purity prior to interpretation of results. Resistance profiles for amikacin, ampicillin-sulbactam, aztreonam, cefepime, cefotaxime, ceftazidime, ceftriaxone, ciprofloxacin, ertapenem, gentamicin, levofloxacin, meropenem, piperacillin, piperacillin-tazobactam, tetracycline, tobramycin, and trimethoprim-sulfamethoxazole were defined. MICs to these antibiotics were calculated by the BD Phoenix instrument software, with interpretations determined according to Clinical and Laboratory Standards Institute (CLSI) M100 guidelines based on the bacterial identification (39).

*P. aeruginosa* strain DSM 1718 (ATCC 27853) and *A. baumannii strain* DSM 1719 (ATCC 19606) were used as comparator strains for their respective species because these strains are commercially available and well characterized. For *P. aeruginosa*, isolates were grown on Lysogeny Broth (LB) agar (Sigma-Aldrich) plates, or in liquid LB (Sigma-Aldrich) for overnight growth and growth curve analysis, or in LB low salt (Sigma-Aldrich) for biofilm assays. *A. baumannii* isolates were also grown in either liquid LB broth or in colonization factor antigen (CFA) media (40). Liquid cultures of both species were grown shaking at 190 rpm at 37°C. Freezing media consisting of LB media + 40% glycerol (EMD Chemicals, Inc.) was used for the creation and storage of −80°C stock cultures.

### Genomic sequencing

Genomic DNA was isolated from all isolates with the Wizard genomic prep kit (Promega) according to the manufacturer’s instructions. The samples were then sent to Seqcenter (seqcenter.com) for genomic sequencing. The sequencing data are deposited in NCBI (Bioproject PRJNA933552). Bioinformatic analyses were performed with VFAnalyzer against the Virulence factors of Pathogenic Bacteria database (VFDB) database (41). Sequence reads for each isolate were assembled using SPAdes v3.15 using “isolate” mode (42). Genome assemblies were quality checked using Quast v5.2.0 (43) and checkM v1.1.3 (44). Assemblies that appeared to contain contaminants or additional organisms were processed through a genome binning workflow with MaxBin v2.2.6 (45) and checkM. All genomes and genome bins were taxonomically classified using GTDB-tk v1.50 and the r202 database. Genomes were annotated for general function using the Patric/BV-BRC system, for virulence factors using the VFDB VFanalyzer (41), and for antibiotic and resistance genes with RGI v6.0.3 (46) against CARDB v4.0.0 (46). A phylogenomic tree was built using Orthofinder v2.5.5 (47) using the Patric/BV-BRC gene calls (48), or the Refseq protein sequences for the existing genomes. For outgroups, NCBI Refseq accessions GCF_000241685.1 (strain AB5075), GCF_026167805.1 (ATCC 17978), GCA_011398515.1 (ATCC 19606), GCF_001936675.2 (DSM30011), GCF_001457535.1 (CIP 70.10), and GCF_047227755.2 (A297) were used for *Acinetobacter*, and GCF_000006765.1 (strain PAO1), GCF_000404265.1 (PA14), GCF_902172305.2 (PAK) and GCF_000258285.1 (ATCC 27853) were used for *Pseudomonas*. Average Nucleotide Identity (ANI) was calculated with fastANI v1.33 and visualized with pheatmap v1.0.12 in R.

### Growth curves

Briefly, 5 mL liquid LB test tube cultures were inoculated with three individual colonies of *P. aeruginosa* or *Acinetobacter* and then grown overnight. The next day, 125 mL flasks containing 12.5 mL of LB media were inoculated with individual overnight cultures to a starting OD_600_ of 0.05 for *P. aeruginosa* or 0.01 for *Acinetobacter*. The cultures were then grown at 37°C, shaking at 190 rpm. OD_600_ readings were taken every hour, and samples were taken for CFU determination every 2 h; these were dilution plated onto LB agar and grown overnight at 37°C. Colonies were enumerated the following day. To help break apart aggregates and to increase the accuracy of OD measurement and CFU enumeration, *Pseudomonas* cultures were sonicated in a Branson 1510 Ultrasonic Cleaner for 15 min.

### Biofilm assays

Biofilms were grown according to the protocols of Merritt et al. (49) with modifications. Briefly, 5 mL liquid LB overnight cultures of *P. aeruginosa* or *Acinetobacter* were inoculated with three individual colonies per isolate and then grown overnight. The following day, these cultures were used to inoculate individual 125 mL flasks containing 12.5 mL of media to a final OD_600_ of 0.05 for *P. aeruginosa* or 0.01 for *Acinetobacter*. These cultures were then allowed to reach the exponential phase by growth for 3 h. At this point, the cultures were OD adjusted to an OD_600_ of 0.1 in LB no salt for the *P. aeruginosa* isolate, or LB or CFA for *Acinetobacter*. Next, 100 μL of the OD-adjusted culture was inoculated per well into a tissue culture-treated 96-well plate (Costar) or peg lid (Innovotech). The plates were then incubated at 37°C for 30 min, 4 h, 24 h, or 48 h without shaking. To determine biomass, the medium was aspirated, the wells were washed with phosphate-buffered saline (PBS; EMD chemicals), dried, and then fixed with methanol (J. T. Baker). Gram’s crystal violet solution (Sigma-Aldrich) that had been diluted to 1% with water was then added to each well; empty control wells served as the blank. Plates were incubated for 15 min, at which point wells were washed with distilled water and air-dried for 5 min at 37°C. The crystal violet was solubilized with differentiation solution (Sigma-Aldrich) for 15 min, and the solubilized crystal violet solution was read at an absorbance of OD_590_ for each sample to quantitate the level of biofilm formation.

To quantitate CFU, a second set of technical replicate wells was aspirated and washed twice with 200 μL of warm sterile PBS to remove any cells remaining from the liquid culture. To remove the cells from the surface of the polystyrene plate, the wells were then filled with 100 μL of PBS, and the sides of the wells were scraped to remove the cells. To further break up cellular aggregates, the 100 μL samples were then supplemented with a final concentration of 2 μM EDTA and sonicated for 10 min in a benchtop water bath (Branson 1510 Ultrasonic Cleaner). The cells were then diluted and plated onto LB agar plates for enumeration after incubation overnight at 37°C.

### Serum exposure

Serum exposure was conducted as previously described (50) with modifications. Briefly, *P. aeruginosa* or *A. baumannii* was grown to early log phase, as described above. The cultures were harvested, and approximately 10^5^ cells were added to 100 μL of PBS, pure human serum (Sigma-Aldrich), or pure human serum that had been heat-inactivated by incubation for 30 min in a 55°C water bath. The cells were gently rocked for 30 min, 1 h, 2 h, or 3 h. The reaction was stopped by the addition of 10 mM EDTA and incubation on ice for 5 min. The cells were next pelleted and washed with PBS and then dilution plated onto LB agar for enumeration. For serum sensitivity in a biofilm, *A. baumannii* biofilms were grown for 48 h as described above. After 48 h, the media were aspirated, and the biofilms were gently washed with sterile PBS. The biofilms were then scraped from the wells, resuspended in PBS, and sonicated for 15 min to break up any remaining aggregates. The biofilm cells were then standardized to an OD_600_ of 0.05, and approximately 10^5^ cells were added to 100 μL of PBS, pure human serum (Sigma), or heat-inactivated serum and incubated for 30 min, 1 h, 2 h, or 3 h. At this point, the cells were pelleted and washed with PBS and dilution plated for enumeration the following day.

### Statistical analysis

Statistical analyses were conducted with Graph-Pad Prism version 6.0g (GraphPad Software, Inc.). A two-way analysis of variance with Dunnett’s multiple comparison correction was used for the analysis of growth curve and biofilm data.

## RESULTS

### Isolation and identification of bacterial isolates

To better understand circulating wound isolates, bacterial isolates were obtained from traumatic wounds as part of the Uniformed Services University Surgical Critical Care Initiative Emory-WounDx study (35); isolates obtained from a total of nine patients were chosen for characterization. Basic epidemiological data for these patients are summarized in Table 1. Briefly, these patients were civilians with an age range of 24–81 years; when grouped by age range, three patients were less than 35 years of age, four were between 40 and 65 years of age, and one was older than 65 years of age. The patients were mostly male (8 of 9) and African American (6 of 9). Traumatic wounds were inflicted by a number of processes (bullet wounds, motorcycle accidents, vehicle crashes, etc.), but the most common form of injury involved some form of vehicle (6 of 9).

**TABLE 1:** Basic epidemiological characteristics of patients from whom *P. aeruginosa* and *Acinetobacter* were obtained

Parameter	Value (% or range)
Median age, years	49 (24–81)
Race/ethnicity	
Black	6 (66)
White	3 (33)
Wound mechanism	Bullet, 2 (22.2)
	Motorcycle accident, 2 (22.2)
	Motor vehicle crash, 2 (22.2)
	Crushed by flying debris, 1 (11.1)
	Pedestrian struck, 1 (11.1)
	Boating accident, 1 (11.1)
Surgical debridement	7 (77.7)
Prior treatment with antibiotics	7 (77.7)

Bacteria were isolated from wound effluent collected during negative-pressure wound therapy applied in the operating room before each surgical debridement. Resulting isolates were identified using the BD Phoenix system, and a subset of isolates was chosen for further study. Due to the prevalence of these species in wounds and tendency for antibiotic resistance, we focused on *P. aeruginosa* (15 isolates) and *Acinetobacter calcoaceticus-baumannii* complex (nine isolates; Table 2). Of these 24 isolates, 3 represented a single isolate obtained from an individual patient, while 21 represented longitudinal isolates obtained from individual patients that were sampled over time. The resistance of these 24 isolates to select antibiotics was identified via the BD Phoenix system (Table 3). Resistance to at least one antibiotic was found for 11 of the 15 *P. aeruginosa* isolates; resistance to the β-lactam aztreonam was the most common, and resistance to fluoroquinolones was the second most common. Seven of the nine *Acinetobacter* isolates demonstrated resistance to a single antibiotic, with piperacillin resistance the most common. Three of the *Pseudomonas* isolates and two of the *Acinetobacter* isolates were sensitive to all antibiotics tested. Given that multidrug resistance is defined as resistance to three or more categories of antimicrobials (51), only the *P. aeruginosa* isolates DSM 1742 and DSM 1746 would be considered multidrug resistant (Table 3).

**TABLE 2:** Isolates used in this study

Lab designation	Description	Accession numbers	Source or reference	Patient number	Wound type/source	Isolation date (mo/day/yr)
DSM 1718	*Pseudomonas aeruginosa* ATCC27853	SAMN44102028	(52)	n/a^*a*^	Clinical material	1971
DSM 1720	*Pseudomonas aeruginosa* isolate, E0641EA2-B	SAMN44102029	This study	64	Crushed by flying debris	4/30/2016
DSM 1721	*Pseudomonas aeruginosa* isolate, E0642WH-A	SAMN44102030	This study	64	Crushed by flying debris	5/28/2016
DSM 1722	*Pseudomonas aeruginosa* isolate, E0641EHON-A	SAMN44102031	This study	64	Crushed by flying debris	5/28/2016
DSM 1723	*Pseudomonas aeruginosa* isolate, E0641EGON-A	SAMN44102032	This study	64	Crushed by flying debris	5/18/2016
DSM 1729	*Pseudomonas aeruginosa* isolate, E0381EAON-A	SAMN44102033	This study	38	Bullet wound	4/15/2015
DSM 1739	*Pseudomonas aeruginosa* isolate, E0641ECON	SAMN44102034	This study	64	Crushed by flying debris	5/8/2016
DSM 1740	*Pseudomonas aeruginosa* isolate, E0161EBON	SAMN44102035	This study	16	Motorcycle accident	1/13/2014
DSM 1741	*Pseudomonas aeruginosa* isolate, E0621WH	SAMN44102036	This study	62	Motor vehicle crash	6/6/2016
DSM 1742	*Pseudomonas aeruginosa* isolate, E0621EHON	SAMN44102037	This study	62	Motor vehicle crash	6/6/2016
DSM 1743	*Pseudomonas aeruginosa* isolate, E0641ED2	SAMN44102038	This study	64	Crushed by flying debris	5/12/2016
DSM 1744	*Pseudomonas aeruginosa* isolate, E0161EAON	SAMN44102039	This study	16	Motorcycle accident	1/10/2014
DSM 1745	*Pseudomonas aeruginosa* isolate, EZJ0061EEON	SAMN44102040	This study	6	Bullet wound	10/13/2012
DSM 1746	*Pseudomonas aeruginosa* isolate, EZJ0061EDON	SAMN44102041	This study	6	Bullet wound	10/10/2012
DSM 1747	*Pseudomonas aeruginosa* isolate, EZJ0061WI	SAMN44102042	This study	6	Bullet wound	11/1/2012
DSM 1748	*Pseudomonas aeruginosa* isolate, EZJ0061EION	SAMN44102043	This study	6	Bullet wound	11/1/2012
						
DSM 1719	*Acinetobacter baumannii* ATCC19606	SAMN44102044	(53)	n/a^*a*^	Urinary tract infection	1948
DSM 1726	*Acinetobacter baumannii/calcoaceticus* complex isolate, E0551EAON-B	SAMN44102045	This study	55	Motor vehicle crash	9/10/2015
DSM 1749	*Acinetobacter baumannii/calcoaceticu*s complex isolate, E0281EDON	SAMN44102046	This study	28	Pedestrian Struck	10/21/2014
DSM 1751	*Acinetobacter baumannii/calcoaceticus* complex isolate, E0511EBON	SAMN44102047	This study	51	Crushed by flying debris	9/27/2015
DSM 1753	*Acinetobacter baumannii/calcoaceticus* complex isolate, E0281EAON	SAMN44102048	This study	28	Motorcycle crash	10/15/2014
DSM 1754	*Acinetobacter baumannii/calcoaceticus* complex isolate, E0281EBON	SAMN44102049	This study	28	Motor vehicle crash	10/17/2014
DSM 1755	*Acinetobacter baumannii/calcoaceticus* complex isolate, E0511EA2	SAMN44102050	This study	51	Motor vehicle crash	9/24/2015
DSM 1756	*Acinetobacter baumannii/calcoaceticus* complex isolate, E0371EA0N	SAMN44102051	This study	37	Motorcycle crash	4/1/2015
DSM 1757	*Acinetobacter baumannii/calcoaceticus* complex isolate, E0642WA	SAMN44102052	This study	64	Motorcycle crash	4/30/2016
DSM 1758	*Acinetobacter baumannii/calcoaceticus* complex isolate, E0511WC	SAMN44102053	This study	51	Motorcycle crash	10/1/2015

^*a*^ n/a, not applicable.

**TABLE 3:** Antibiotic resistance profile of clinical isolates^*a*^

Antibiotic category	Aminoglycoside			β-lactam			β-lactam plus β-lactamase inhibitor		Monobactam	Carbapenem	Cephalosporin			Folate pathway inhibitors	Fluoroquinolone		Tetracycline
Antibiotic	Amikacin	Gentamicin	Tobra-mycin	Cefotaxime	Meropenem	Pipera-cillin	Ampicillin-Sulbactam	Piperacillin-Tazobactam	Aztreonam	Ertapenem	Cefepime	Ceftazidime	Ceftriaxone	Trimethoprim-Sulfamethoxazole	Ciproflo-xacin	Levoflox-acin	Tetracyc-line
Strain																	
DSM 1720	≤8 S	≤2 S	≤2 S		2 S			16/4 S	>16 R		8 S	8 S			≤0.5 S	2 S	
DSM 1721	≤8 S	≤2 S	≤2 S		2 S			16/4	>16 R		8 S	8 S			≤0.5 S	2 S	
DSM 1722	≤8 S	≤2 S	≤2 S		4 I			32/4 I	>16 R		16 R	16 I			≤0.5 S	2 S	
DSM 1723	≤8 S	≤2 S	≤2 S		2 S			16/4 S	>16 R		8 S	8 S			≤0.5 S	2 S	
DSM 1729	≤8 S	≤2 S	≤2 S		≤1 S	8 S		8/4 S	8 S		2 S	2 S			≤0.5 S	≤1 S	
DSM 1739	≤8 S	≤2 S	≤2 S		2 S			16/4 S	>16 R		8 S	4 S			≤0.5 S	2 S	
DSM 1740	≤8 S	≤2 S	≤2 S		≤1 S	8 S		4/4 S	8 S		4 S	2 S			≤0.5 S	≤1 S	
DSM 1741	≤8 S	≤2 S	≤2 S		≤0.5 S			16/4 S	4 S		8 S	4 S			>2 R	4 I	
DSM 1742	≤8 S	≤2 S	≤2 S		4 I			>64/4 R	>16 R		>16 R	>16 R			1 S	2 S	
DSM 1743	≤8 S	≤2 S	≤2 S		2 S			16/4 S	>16 R		8 S	8 S			≤0.5 S	≤1 S	
DSM 1744	≤8 S	≤2 S	≤2 S		≤1 S	≤4 S		4/4 S	8 S		2 S	1 S			≤0.5 S	≤1 S	
DSM 1745	≤8 S	≤2 S	≤2 S		2 S	32 S		32/4 S	>16 R		16 I	8 S			>2 R	>4 R	
DSM 1746	16 S	4 S	≤2 S		>8 R	>64 R		64/4 S	>16 R		16 I	8 S			>2 R	>4 R	
DSM 1747	≤8 S	≤2 S	≤2 S		4 S	32 S		32/4 S	>16 R		16 I	8 S			>2 R	>4 R	
DSM 1748	≤8 S	≤2 S	≤2 S		2 S	32S		32/4 S	>16 R		16 I	8 S			>2 R	>4 R	
DSM 1726	≤8 S	4 S	≤2 S	16 I	≤1 S	32 I					4 S	4 S	16 I	1/19 S	≤0.5 S	≤1 S	
DSM 1749	≤8 S	4 S	≤2 S	8 S	≤1 S	16 S					≤1 S	4 S	4 S	≤0.5/9.5 S	≤0.5 S	≤1 S	
DSM 1751	≤8 S	≤2 S	≤2 S	8 S	≤1 S	16 S					2 S	8 S	4 S	≤0.5/9.5 S	≤0.5 S	≤1 S	
DSM 1753	≤8 S	4 S	≤2 S	32 I	≤1 S	R					8 S	8 S	16 I	≤0.5/9.5 S	≤0.5 S	≤1 S	
DSM 1754	≤8 S	≤2 S	≤2 S	32 I	≤1	R					8 S	16 I	32 I	≤0.5/9.5 S	≤0.5 S	≤1 S	
DSM 1755	≤8 S	≤2 S	≤2 S	8 S	≤1 S	R					≤1 S	4 S	8 S	≤0.5/9.5 S	≤0.5 S	≤1 S	
DSM 1756	≤8 S	≤2 S	≤2 S	8 S	≤1 S	R					≤1 S	4 S	8 S	≤0.5/9.5 S	≤0.5 S	≤1 S	
DSM 1757	≤8 S	≤2 S	≤2 S		≤0.5 S		2/1 S			R	2 S	4 S	16 I	≤0.5/9.5 S	≤0.5 S	≤1 S	≤2 S
DSM 1758	≤8 S	≤2 S	≤2 S	16 I	≤1 S	R					4 S	8 S	8 S	≤0.5/9.5 S	≤0.5 S	≤1 S	

^*a*^ ≤ indicates less than or equal to the given value, while > indicates greater than the given value. Numbers indicate a given concentration of drug in µg/ml as the AST standard. S, sensitive; I, intermediate; R, resistant, based on CLSI M100 cutoffs for each identified bacterium. Cells containing R were determined to be resistant but no value was returned. Similarly, empty cells indicate no data for that particular drug/isolate.

Differences in the antibiotic resistance profiles of isolates obtained longitudinally from the same patient were also noted. For *Acinetobacter*, the final isolate from patient 28 was more sensitive to piperacillin, ceftriaxone, and cefepime than the first isolate obtained from this patient. *P. aeruginosa* longitudinal isolates obtained from patients 6 and 64 also showed temporal variation in antibiotic resistance. Later isolates from patient 6 displayed resistance to the β-lactam antibiotic aztreonam and to the fluoroquinolones ciprofloxacin and levofloxacin but sensitivity to the β-lactams meropenem, piperacillin, and piperacillin-tazobactam. For patient 64, the later *P. aeruginosa* isolates showed resistance to meropenem, piperacillin-tazobactam, cefepime, and ceftazidime. Utilization of specific antibiotics within a patient often correlated with the occurrence of resistance in the analyzed isolates. Specifically, patient six was treated with bacitracin, ciprofloxacin, and erythromycin; resistance to bacitracin and erythromycin was not investigated as part of the BD Phoenix panel. Similarly, patient 64 was treated with cefepime and cefazolin, creating selective pressure for β-lactam and cephalosporin resistance. Thus, the variation in observed antibiotic resistance across isolates may represent selective pressure from the chosen treatment regime used for that particular patient.

### Genotypic characterization of isolates

Given that *A. baumannii* is noted for multidrug resistance in both clinical (18) and environmental settings (54), the lack of multidrug resistance in the analyzed *Acinetobacter* isolates was unexpected. This raised the possibility that some of the *Acinetobacter* isolates were not *A. baumannii. A. baumannii* belongs to the *Acinetobacter calcoaceticus-baumannii* complex, which consists of the closely related species of *Acinetobacter baumannii*, *A. calcoaceticus*, *A. nosocomialis*, *A. pittii* (55), *A. seifertii* (56), *A. dijkshoorniae* (57), *A. geminorum* (58), and *A. oleivorans* (59, 60). It can be difficult to differentiate among the closely related species in the *A. calcoaceticus-baumannii* complex, even using automated systems like the BD Phoenix system or matrix-assisted laser desorption/ionization with time of flight (8, 61–65). Accurate species identification often requires sequencing of the 16S ribosomal subunit, or multi-locus sequence typing (MLST) to differentiate to the species level; even with these methods, accurate speciation is still difficult (65). Nonetheless, correct identification of the particular species within the *A. calcoaceticus-baumannii* complex is important because infection with *A. baumannii* is more strongly associated with poor clinical outcomes than other members of the complex (66). To define the species of the *Acinetobacter* wound isolates, as well as to define genetic components of both *Acinetobacter* and *P. aeruginosa* clinical isolates, all 24 wound isolates and two commonly used reference strains (ATCC 27853 for *P. aeruginosa* and ATCC 19606 for *A. baumannii*) were subjected to whole-genome sequencing. The annotated genomic sequences are available through NCBI Bioproject PRJNA933552 and individually under the accession numbers provided in Table 2. Sequencing revealed that the average size of the *P. aeruginosa* genome was 6,616,000 bp compared to the reference genome’s 6,817,428 bp. For *Acinetobacter*, the average genome size was 4,024,000 bp compared to the reference genome’s 3,959,000 bp. To confirm that the sequencing results obtained here were comparable to the published sequences for ATCC27853 and ATC19606, breseq was utilized to identify nucleotide differences between the genomes; only 11 and 26 base substitutions were identified in the re-sequencing of the ATCC strains for *Pseudomonas* and *Acinetobacter*, respectively. This small number of nucleotide changes suggests little lab evolution of these strains as compared to the resequenced isolates (Table S3).

As highlighted above, identification of a particular species within the *A. calcoaceticus-baumannii* complex can be challenging and is affected by the chosen method or analyzed genes. For example, the BD Phoenix identification revealed that all of the isolates were either *A. baumannii*, *A. calcoaceticus-baumannii* complex, or an *Acinetobacter* species. However, results of sequencing-based species identification are dependent on the particular genes analyzed. As a specific example of the complexity of species identification, isolate DSM 1749 was identified by the BD Phoenix system as an *Acinetobacter* species. Post-genome sequencing, a blast search of the genes used for MLST typing (*gltA*, *gyrB*, *gdhB*, *recA*, *cpn60*, *gpi*, and *rpoD*) (67) indicated that the isolate might be *A. courvalinii*. However, analysis of the *rpoB*, *bla* genes, and 16S ribosomal subunit sequence indicated that the isolate might be *A. vivianii*, *A. courvalinii*, or *A. baumannii*. Furthermore, the NCBI portal identified the isolate as *A. calcoaceticus* upon analysis of the entire uploaded genome. These differing results underscore the current difficulty in clearly defining a particular *Acinetobacter* species. It is clear that these clinical isolates are members of the *A. calcoaceticus-baumannii* complex, but it is difficult to define an exact species. Given this, hereafter, these isolates were collectively referred to as *Acinetobacter*.

The annotated genome sequences were next compared to the VFDB (68) and the Comprehensive Antibiotic Resistance Database (CARD) (46); these analyses resulted in pathway resolution of known virulence factors and resistance genes (Tables S1 and S2). In keeping with the unexpected lack of multi-drug resistance displayed by the *Acinetobacter* isolates as determined by the BD Phoenix system, none of the *Acinetobacter* genomes showed any hits in the CARD database. In contrast, many of the *P. aeruginosa* isolates carried genes that are known to encode factors associated with antibiotic resistance; these included the genes encoding resistance-nodulation-cell division and major facilitator superfamily antibiotic efflux pumps, sulfonamide resistance (*sul1* or *sul2*, depending on the strain), and fluoroquinolone resistance (*gyrA*; Table S1) genes as well as nucleotide changes that did not appear in the reference strain ATCC 27853.

For longitudinal isolates from individual patients, sequencing did not reveal the acquisition of known new antibiotic resistance (or virulence genes), suggesting that observed isolate differences in antibiotic resistance were likely due to mutations in existing genes. For example, we noted a single amino acid change in *mexA* that appeared to correlate with meropenem sensitivity in the *P. aeruginosa* isolates. Similarly, isolates that displayed meropenem and ceftazidime resistance all appeared to have lost the *nalD* gene that encodes a repressor of MexAB-OpmR (69). Among *Acinetobacter* isolates that were sensitive to piperacillin and/or ceftriaxone, the loss of an efflux pump (*abaQ*) (70) or a gene encoding a protein responsible for enzymatic degradation of antibiotics (ANT(3″)-IIc) (71) was observed. Notably, these genes are not known to be directly involved with resistance to these two specific antibiotics, and these findings may represent an avenue for future exploration into antibiotic resistance in this genus. For the isolates that displayed resistance to the β-lactam cefotaxime, these isolates appeared to contain alleles of Acinetobacter-derived cephalosporinases that degrade β-lactams (72, 73). Additionally, only 11 and 26 base substitutions were identified in the re-sequencing of the ATCC strains for *Pseudomonas* and *Acinetobacter*, respectively, further suggesting little lab evolution of these strains compared to the sequenced isolate (Table S3).

In addition to potential changes within antibiotic resistance genes, comparisons of the sequenced genomes of the clinical isolates to the reference isolates revealed potential changes in the presence of genes (Table S2). Eleven of the 15 *P. aeruginosa* clinical isolates did not carry an annotated *pilA* gene, which encodes the basic subunit used to build pili (74). Of those isolates, five also appeared to be missing *fimT* and *pilY2*, which are involved in the assembly of pili. Other mutations or missing genes were found to include *wcaJ*, which encodes a protein known to be involved in modification of the lipopolysaccharide core unit in *Escherichia coli* (75) and phospholipase D. Altered pathways included phenazine production (76), alginate regulation, capsular polysaccharide, the *mymA* operon, pyoverdine production, the Type IV secretion system, the *icm*/*dot* type IVB secretion system, and Type 1 fimbriae, all of which could play a role in biofilm formation. *Acinetobacter* genes/components that were potentially altered included the *acrAB* (bacillibactin and mycobactin) genes, as well as genes encoding components involved in heme utilization, LPS, and Poly-N-acetyl glucosamine and quorum-sensing pathways.

**Fig 1:**
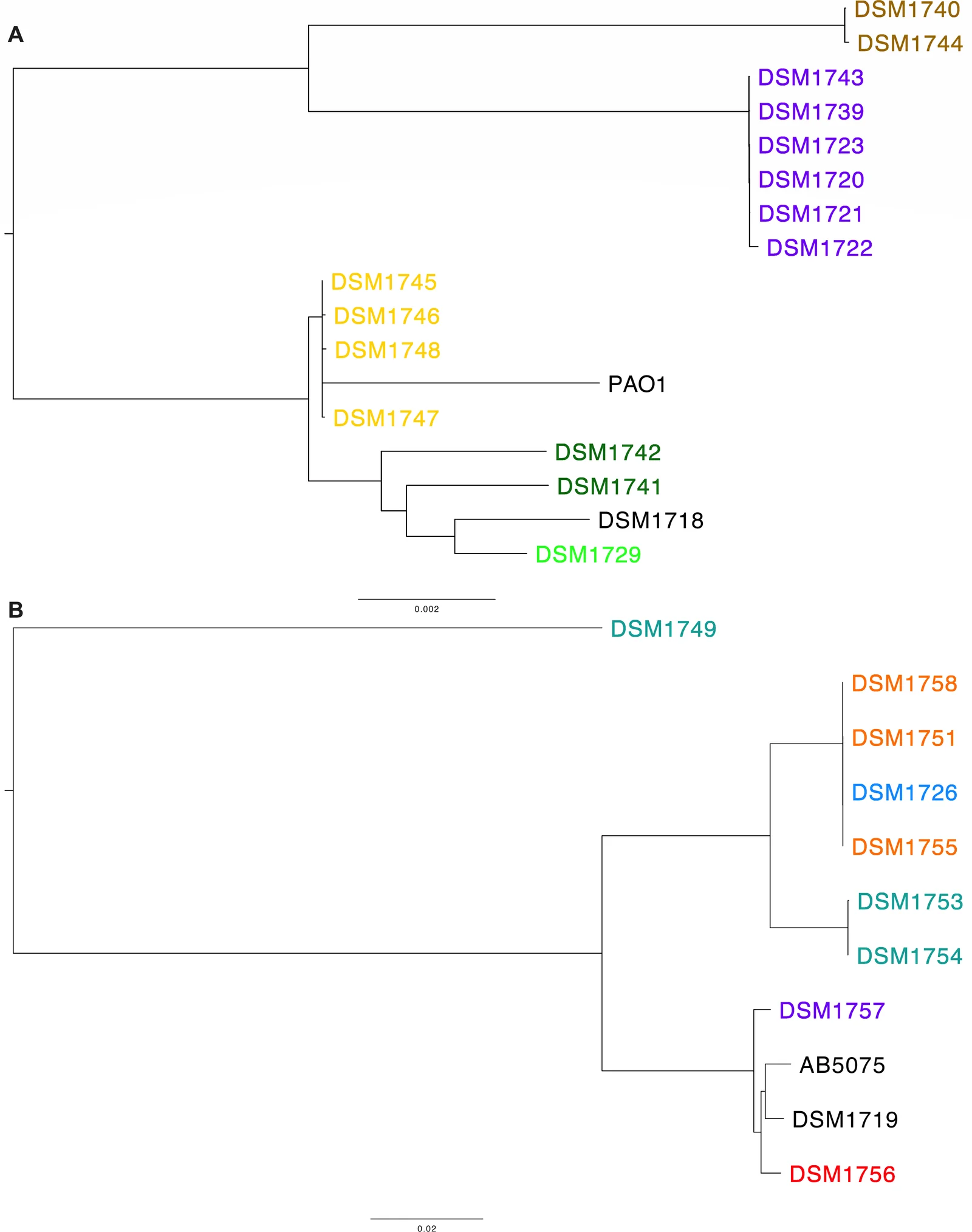
Phylogenetic relationship of *P. aeruginosa* and *Acinetobacter* clinical isolates and reference strains. Species trees were constructed from 4,461 and 1,954 conserved, single-copy genes for *P. aeruginosa* (**A**) and *Acinetobacter* (**B**), respectively. Isolates obtained from the same patient are denoted by the use of the same color for the strain name designations. Reference strains are colored in black. Branch length and scale bar refer to the mean amino acid substitutions per site (e.g., branch length of 0.02 is equal to 1 amino acid change per 50 sites).

To determine the overall relatedness of the various isolates, individual phylogenetic trees were assembled based on single copy orthologs: 4,461 genes for *P. aeruginosa* or 1,954 genes for *Acinetobacter*. The common laboratory strains PAO1 (*P. aeruginosa*) or AB5075 (*A. baumannii*), as well as the appropriate ATCC reference strain, were included for each (Fig. 1). For both species, the isolates from the same patient grouped closely together, suggesting that these isolates are likely closely related. Initially, we expected the laboratory isolates to be separated from all of the clinical isolates; however, these isolates, which were isolated many decades ago and serially passaged within the laboratory, grouped closely with clinical strains that were isolated more recently. This suggests that these highly utilized laboratory strains remain closely related to newer clinical isolates when considering the whole genome. Moreover, similar results were found even when the phylogenetic tree was expanded to include additional wound isolates (Fig. S3).

**Fig 2:**
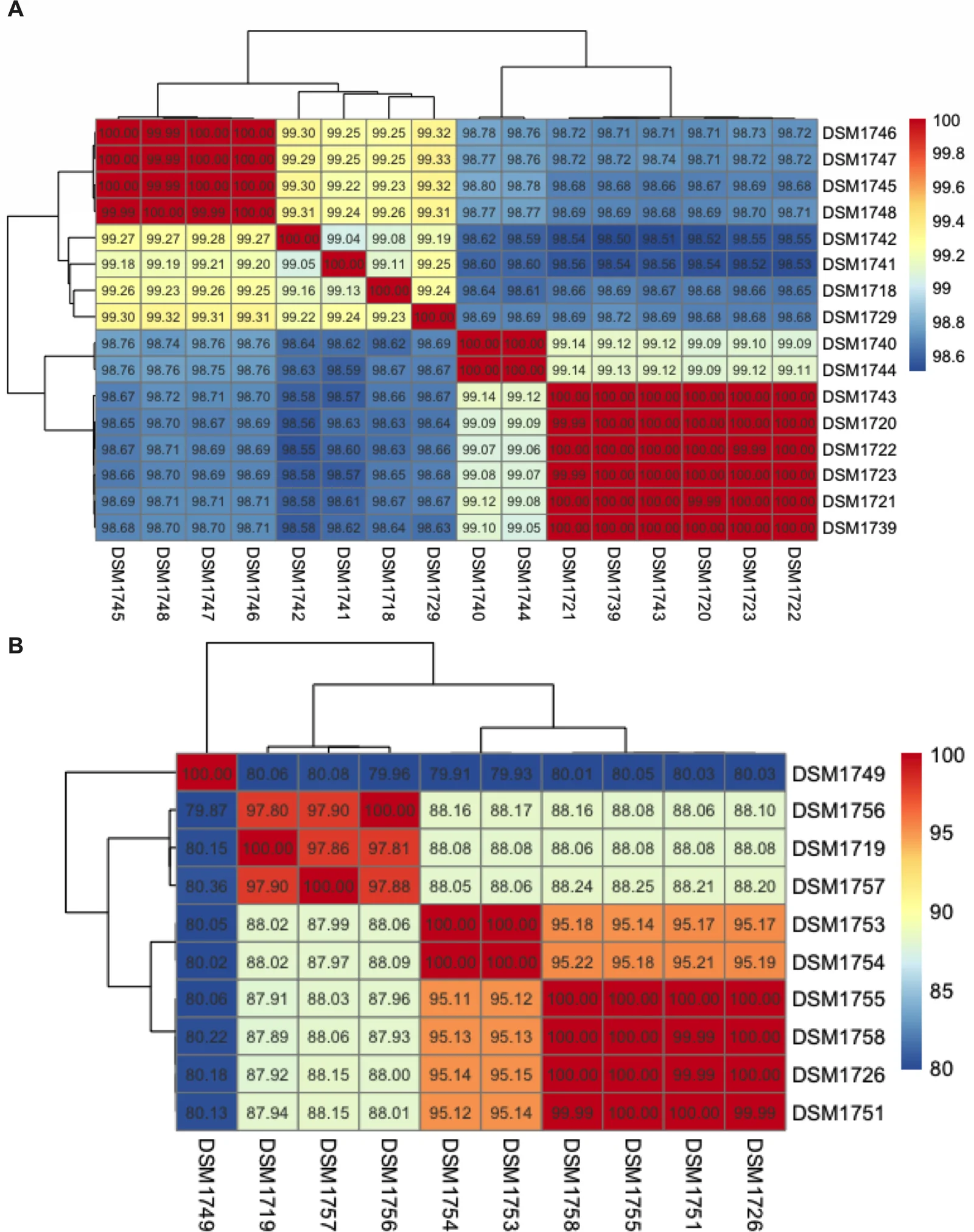
Average nucleotide identity (ANI) analysis to determine isolates’ relatedness. ANI analysis was performed on the indicated (**A**) *P. aeruginosa* and (**B**) *Acinetobacter* isolates. The correlation matrix’s heatmap was used to analyze the variations among the different isolates, with the scale going from dissimilar (blue) to similar (red). Dendrograms represent “complete” clustering from the pheatmap R package. ANI values equal to or greater than 99.9% were considered identical isolates.

Given that some of these isolates were collected longitudinally from the same patients, we next utilized ANI with a cutoff of 99.9% to determine whether any of the individual isolates were identical (Fig. 2). This analysis revealed that the isolates from the same patient were so highly similar to one another that they could be considered the same. For example, the *P. aeruginosa* isolates DSM 1722, 1723, 1720, 1743, 1739, and 1721 were all from the same patient and showed a high degree of similarity (Fig. 2). Likewise, DSM 1744 and 1740 were isolated from the same patient and appeared to be the same. The same was true for DSM 1746, 1747, 1748, and 1745, which were all isolated from the same patient.

The ANI results for the *Acinetobacter* isolates were more complex. For example, DSM 1726, 1751, 1755, and 1756 were identical isolates according to the utilized criteria. However, DSM 1726 was isolated from a different patient than the other three isolates. Similarly, DSM 1753 and 1754 that came from the same patient were highly similar and considered the same. However, DSM 1749 was isolated from this same patient but was only distantly related to all of the other *Acinetobacter* isolates, including the ones from the same patient. This distance led us to reanalyze the 16S ribosomal sequence and MLST profile for this isolate, both of which indicated that this strain is a member of the *A. calcoaceticus-baumannii* complex. Overall, the ANI results indicate that most of the isolates from the same patient can be considered the same.

### The variable biofilm formation of clinical *Pseudomonas* and *Acinetobacter* isolates

**Fig 3:**
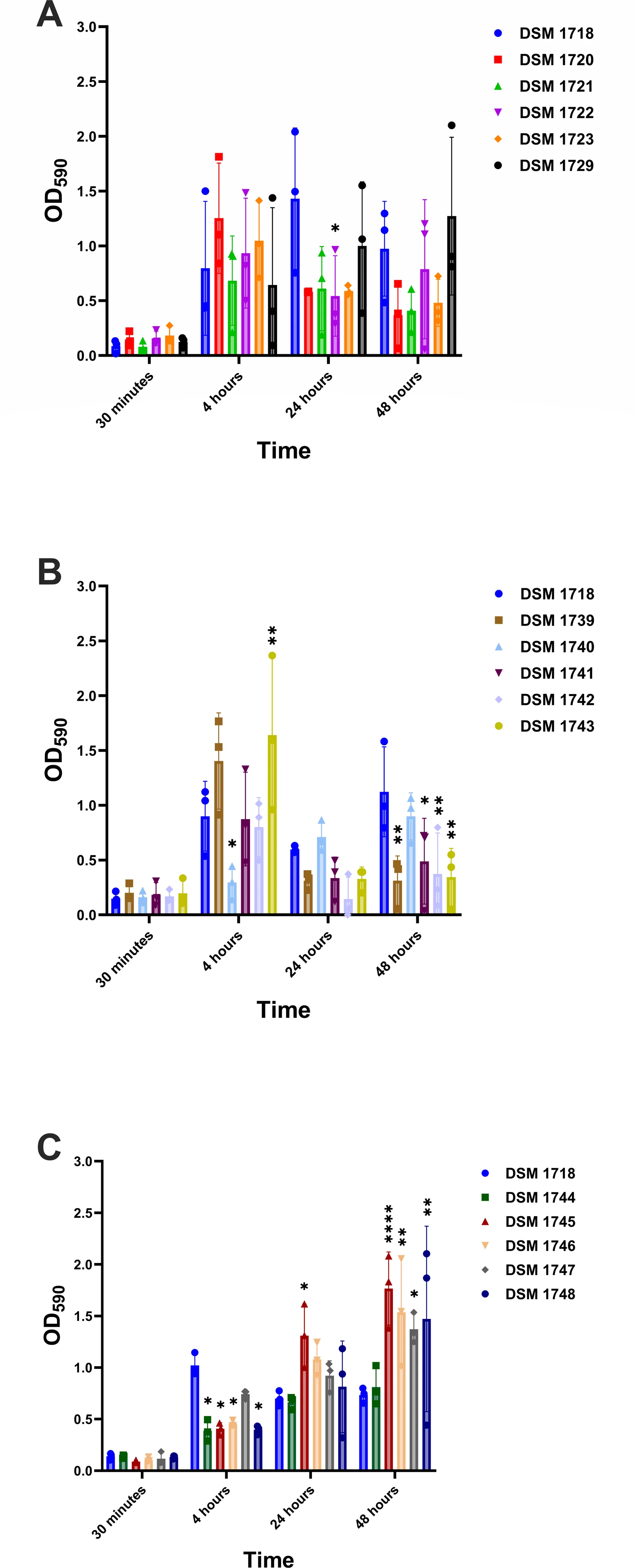
*Pseudomonas aeruginosa* biofilm formation. Strains of *P. aeruginosa* were grown in a 96-well plate biofilm format for 30 min, 4 h, 24 h, and 48 h. At each time point, the biofilm biomass was quantified by crystal violet staining followed by solubilization and measurement of the OD_590_. In each panel, independent data for strain DSM 1718 are included for direct comparison to the clinical isolates also represented within that panel. (**A**) Strains DSM 1718, DSM 1720–1723, and 1729. (**B**) Strains DSM 1718 and DSM 1739–1743. (**C**) Strains DSM 1718 and DSM 1744–1748. Data were obtained from three biologically independent replicates; individual data points and the mean are plotted; error bars display standard deviation. Two-way analysis of variance with Dunnett correction for multiple comparisons was performed; *P < 0.05, **P < 0.01, and ****P < 0.0001 for the indicated isolate as compared to DSM 1718 at that timepoint.

**Fig 4:**
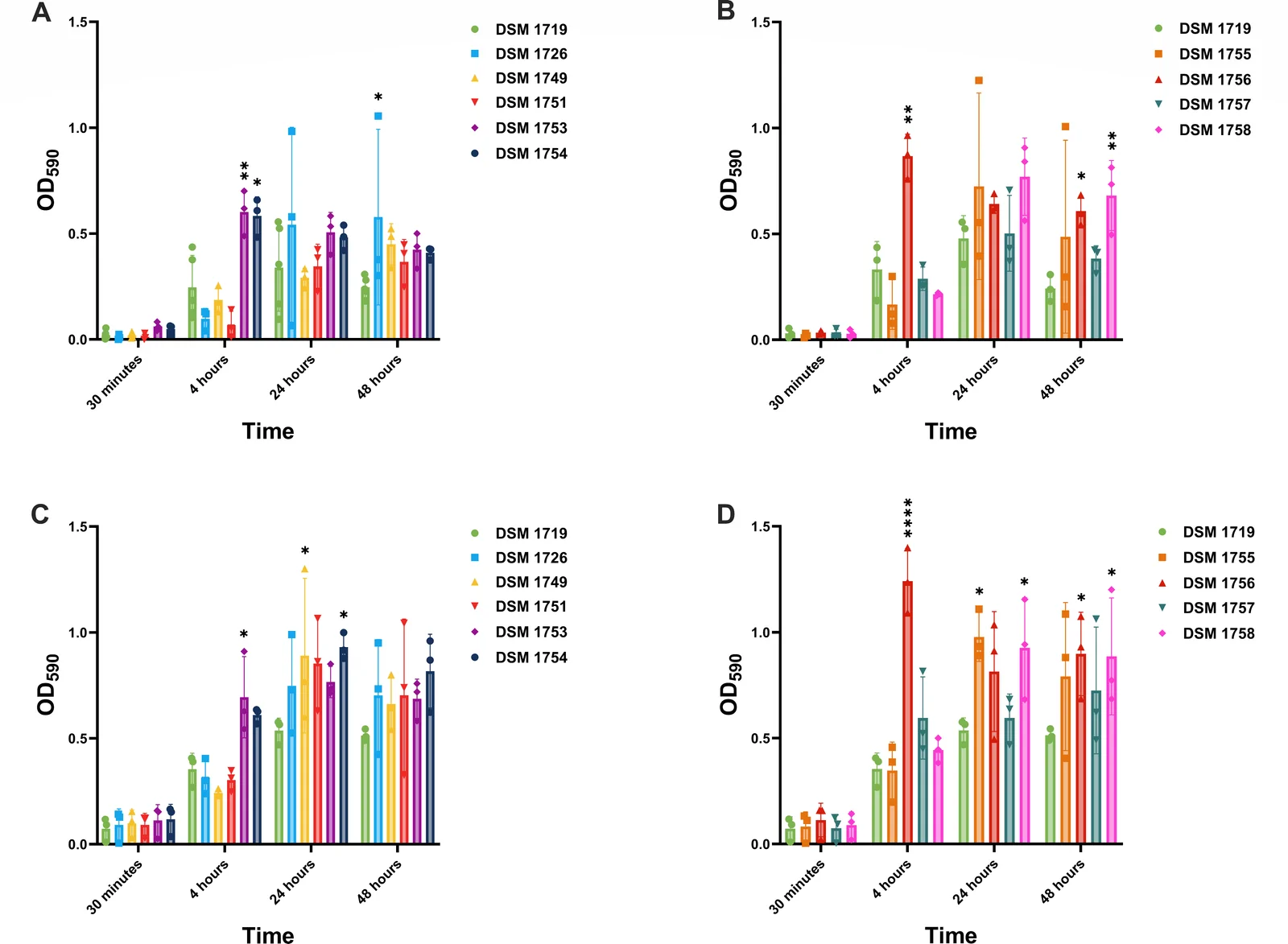
*Acinetobacter* biofilm formation. Isolates of *Acinetobacter* were grown in a biofilm 96-well plate format for 30 min, 4 h, 24 h, and 48 h in either LB media (**A and B**) or CFA media (**C and D**). At each time point, the biofilms were quantified by crystal violet staining followed by solubilization and reading of the OD_590_. In each panel, independent data for strain DSM 1719 are included for direct comparison to the clinical isolates also represented within that panel. (**A and C**) Strains DSM 1719, 1726, 1749, 1751, 1753, and 1754. (**B and D**) Strains DSM 1755–1758. Data were obtained from at least three biologically independent replicates; individual data points and the mean are plotted; error bars display standard error of the deviation. Two-way analysis of variance with Dunnett correction for multiple comparisons was performed; *P < 0.05, **P < 0.01, and ****P < 0.0001 for the indicated isolate as compared to DSM 1719 at that timepoint.

Given the identified potential differences in annotated genes that affect biofilm formation, all of the clinical and reference isolates were next characterized for the ability to form biofilms. To this end, the isolates were grown in LB media in tissue culture-treated 96-well plates, and biofilm formation was assessed at 30 min, 4 h, 24 h, and 48 h by crystal violet staining. This assay demonstrated variability in the timing and magnitude of biofilm formation by the *P. aeruginosa* isolates (Fig. 3). Many of these isolates hit maximal biofilm growth at 4 h, while others slowly reached their maximum biofilm formation at 48 h. The *Acinetobacter* isolates also demonstrated similar isolate-dependent variation in biofilm formation timing and magnitude when grown in LB media (Fig. 4A and B). Given that CFA medium (40) has been shown to promote biofilm formation by *Acinetobacter* (77), we also assessed biofilm formation using this medium instead of LB. When grown in CFA media, the *Acinetobacter* isolates tended to achieve a higher biomass than when grown in LB media (compare Fig. 4A through D). However, the general trends for timing and magnitude of biofilm formation per *Acinetobacter* isolate remained the same regardless of the media used.

**Fig 5:**
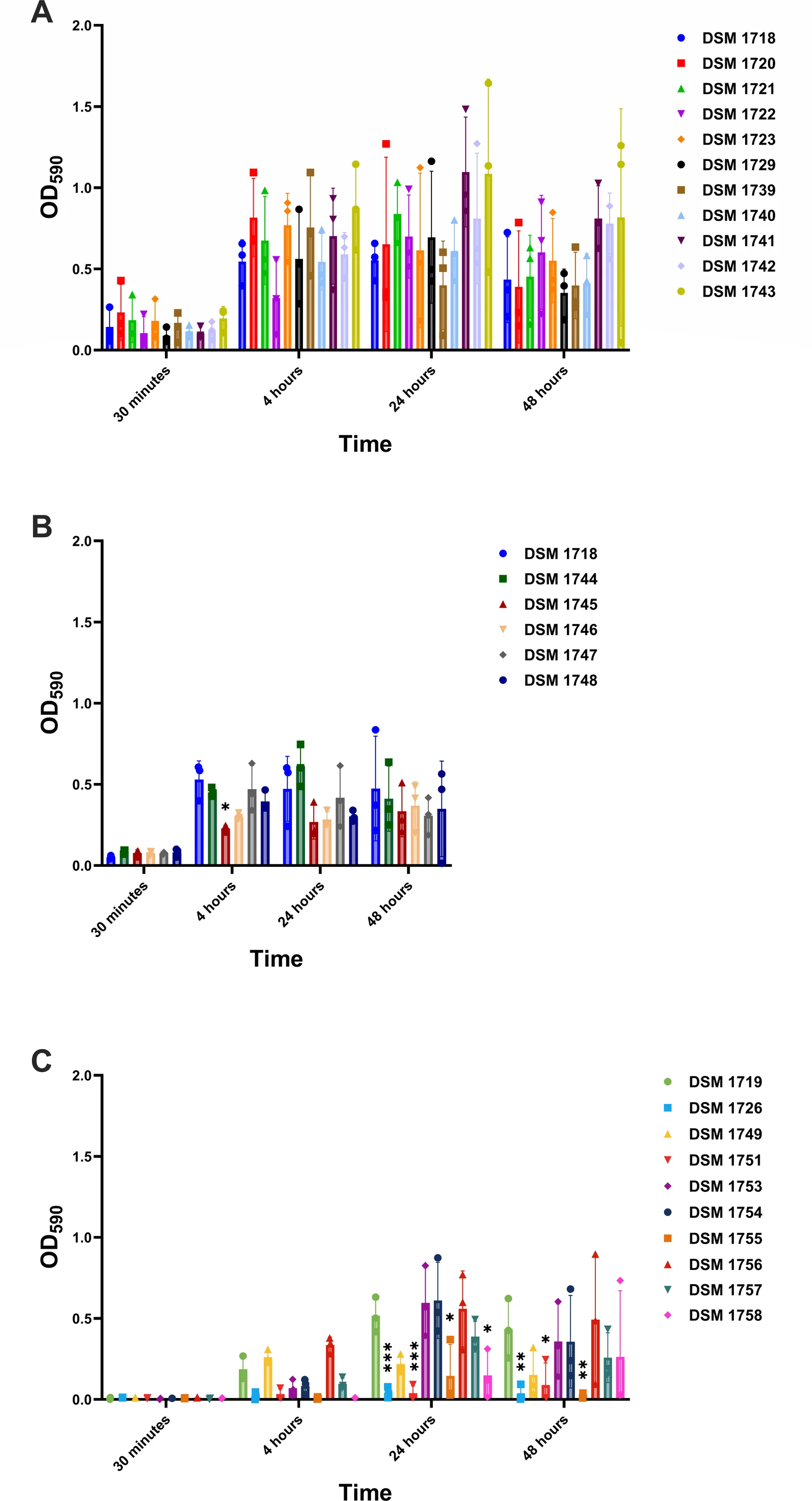
*P. aeruginosa* and *Acinetobacter* biofilm formation in a peg lid plate. Isolates of *P. aeruginosa* and *Acinetobacter* were grown in a peg lid plate biofilm for 30 min, 4 h, 24 h, and 48 h. At each time point, the biofilms were quantified by crystal violet staining followed by solubilization and measurement of the OD_590_. In panels A and B, independent data for strain DSM 1718 are included for direct comparison to the clinical isolates also represented within that panel. (**A**) *P. aeruginosa* isolates DSM 1720–1723, 1729, and 1739–1743, (**B**) *P. aeruginosa* strains DSM 1744–1748, and (**C**) all *Acinetobacter* isolates. Data were obtained from three biologically independent replicates; individual data points and the mean are plotted; error bars display standard deviation. Two-way analysis of variance with Dunnett correction for multiple comparisons was performed; *P < 0.05, **P < 0.01, and ***P < 0.001 for the indicated strain as compared to DSM 1718 for *P. aeruginosa* (**A and B**) or DSM 1719 for *Acinetobacter* (**C**) at that timepoint.

In addition to growth in a 96-well plate, biofilm formation in a peg lid format was also tested (Fig. 5). In this format, the *P. aeruginosa* isolates demonstrated less variation and less robust growth (Fig. 5A and B). *Acinetobacter* demonstrated more variation but also reduced biofilm formation than when grown in 96-well plates. While this may be due to differences in plate material, the decrease in biofilm growth may also be attributed to the fact that the surface area of the 96-well plate is larger than the surface area of the peg lid. Growth curve analysis revealed that only isolates DSM 1745 and DSM 1757 demonstrated statistically significant differences in both OD and CFU compared to their respective reference strains (Fig. S1). Only in the case of DSM 1745 did an increased growth rate correlate with increased biofilm (Fig. 3C). Therefore, the differences between these isolates in all of the biofilm assays cannot be wholly attributed to any obvious differences in growth.

### Biofilm formation protects some *Acinetobacter* isolates from serum-mediated killing

Existence within a biofilm can protect bacterial cells from the immune system (26–29) and has been intricately linked to virulence for numerous bacterial pathogens (53, 78–80). Given this, a subset of the isolates (five *P. aeruginosa* isolates and five *Acinetobacter* isolates) was selected to determine if biofilm formation would protect cells from human serum-mediated killing as compared to planktonically grown cells. However, all of the tested planktonically grown *P. aeruginosa* isolates were fully resistant to human-serum mediated killing (Fig. S2). Therefore, planktonic versus biofilm comparisons could only be conducted with *Acinetobacter*.

**Fig 6:**
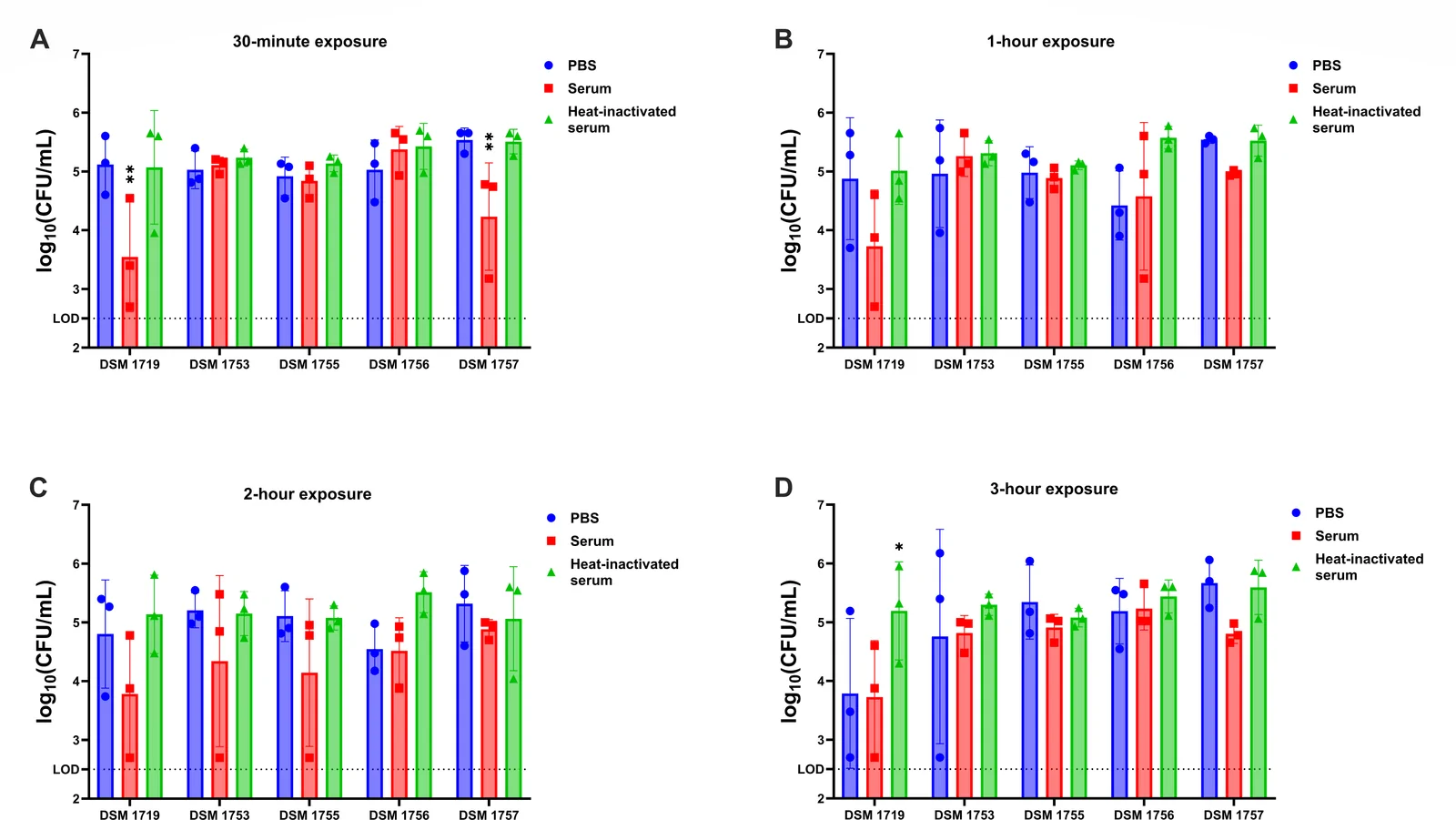
Serum resistance profiles of *Acinetobacter* cells from liquid culture. Cultures of *Acinetobacter* isolates were grown for 3 h and OD adjusted to OD_600_ of 0.05. Next, 2.5 μL of this adjusted culture (~10^5^ cells) were added to serum, PBS, or heat-inactivated serum for (**A**) 30 min, (**B**) 1 h, (**C**) 2 h, or (**D**) 3 h; and the surviving CFU were enumerated and log-transformed for plotting and analysis. Data were obtained from three biologically independent replicates; individual data points and the mean are plotted; error bars display standard deviation; LOD, limit of detection. Two-way analysis of variance with Dunnett correction for multiple comparisons was performed; *P < 0.05 and **P < 0.01 as compared to the PBS control for that strain at that timepoint.

Exposure of liquid-grown planktonic cells to human serum for 30 min significantly reduced the recovered CFU from the *Acinetobacter* reference isolates (DSM 1719) and the clinical isolate DSM 1757 (Fig. 6A). In contrast, incubation with PBS or heat-inactivated serum did not result in a change in recovered CFU for these or any other isolates; these data indicate that the observed decrease for DSM 1757 and the reference strain represents serum-mediated killing. Similar CFU decreases for DSM 1719 and DSM 1757 were noted after 1, 2, and 3 h of serum exposure, though these decreases were not statistically significant for these or any of the other isolates (Fig. 6B and C). It was noted that the reference strain DSM 1719 demonstrated a statistically significant increase in CFU when exposed for 3 h to heat-inactivated serum, suggesting that this strain has the ability to support growth using the nutrient-rich inactivated serum.

**Fig 7:**
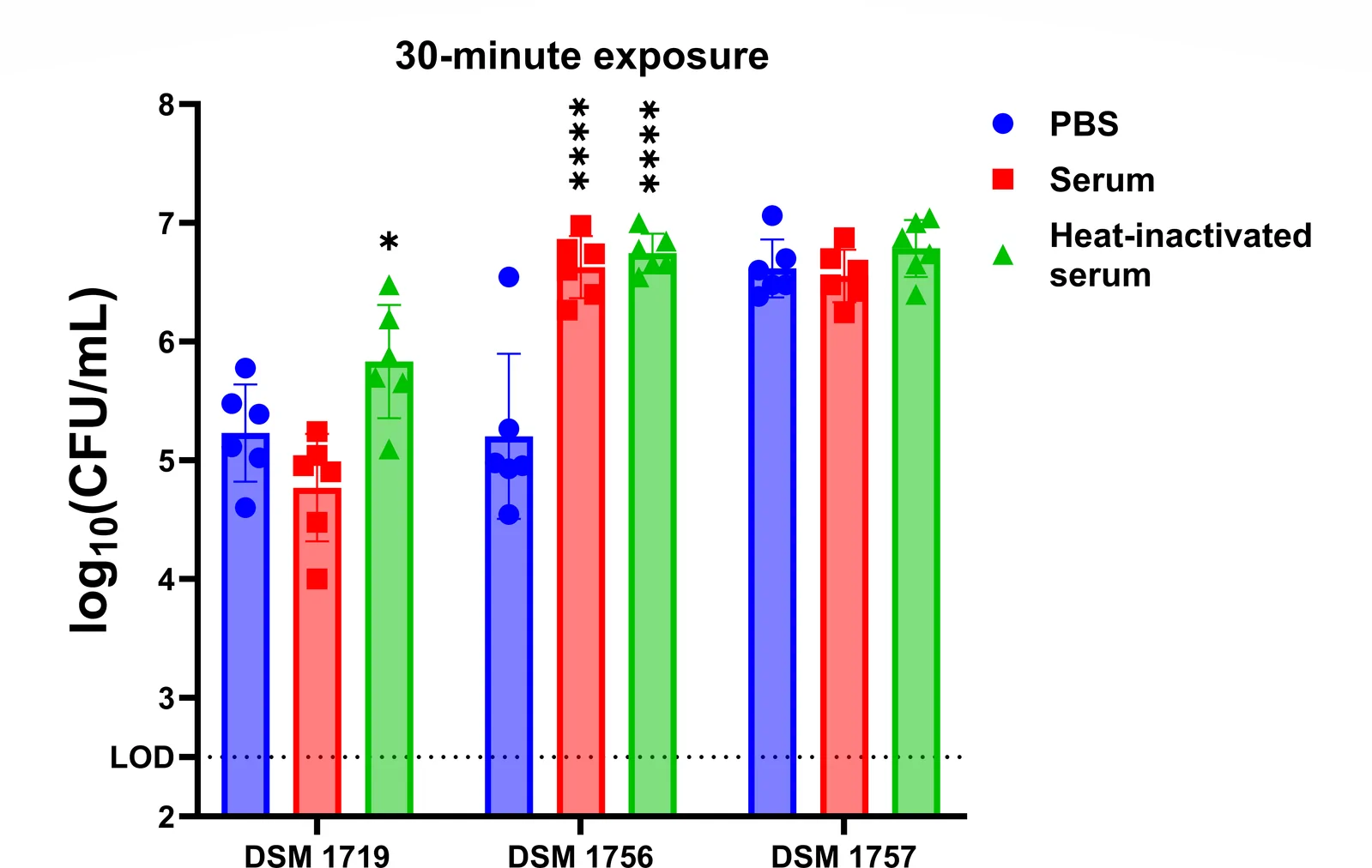
Serum resistance of *Acinetobacter* cells grown in biofilm. Isolates of *Acinetobacter* were grown in a biofilm for 48 h. The media were aspirated, the biofilm washed, and the cells scraped from the well and resuspended in PBS. The resuspension was further sonicated to break apart any aggregates. The cells were then resuspended in either PBS, serum, or heat-inactivated serum for 30 min, after which the cells were washed; surviving CFU were enumerated and log-transformed for plotting and analysis. Data were obtained from six biologically independent replicates; individual data points and the mean are plotted; error bars display standard deviation; LOD, limit of detection. A two-way analysis of variance with Dunnett correction for multiple comparisons was performed; *P < 0.05 and ****P < 0.0001 as compared to the PBS control for that isolate.

Given the planktonic sensitivity of the reference strain and DSM 1757 to heat-inactivated serum, these two strains were next assessed to determine if biofilm growth would alter serum-susceptibility of these isolates; DSM 1756 was included as a control since it was resistant to serum-mediated killing (Fig. 6). The biofilms were grown for 48 h, the liquid media was aspirated, and then the biofilm was disrupted, and these cells were incubated with serum. As shown in Fig. 7, none of the tested biofilm-grown strains showed a reduction in CFU when exposed to serum. This result suggests that biofilm-grown cells express factors that protect them from serum-mediated killing, even once the biofilm matrix is disrupted. We also noted that DSM 1719 once again seemed to be able to use heat-inactivated serum to support growth, and biofilm grown DSM 1756 was able to grow in serum regardless of whether the serum had been heat inactivated. Overall, these data suggest that biofilm growth can result in serum protection for some *Acinetobacter* strains, even after release from the biofilm matrix.

## DISCUSSION

To better understand circulating strains that infect traumatic wounds, here we characterized clinical isolates of *P. aeruginosa* and members of the *A. calcoaceticus-baumannii* complex isolated from traumatic wounds at Emory University Hospital. Analysis of the antibiotic resistance profile of these isolates, as measured using the BD Phoenix system, revealed that a small subset of the isolates was multidrug resistant (Table 2); however, isolates that were obtained from patients at later dates seemed to show an evolution of antibiotic resistance profiles that often correlated with antibiotic treatment regimes used for that patient. Phylogenetic analysis revealed that longitudinal isolates from the same patient tended to group closely with isolates obtained from the same patient (Fig. 1; Fig. S3). Additionally, some of these isolates grouped closely to commonly utilized laboratory strains. Moreover, ANI comparison revealed that isolates from the same patient were similar enough to be considered the same isolate (Fig. 2). Though there were temporal and magnitude differences, all isolates formed biofilms under the tested conditions (Fig. 3 to 5). Additionally, only one of the tested clinical isolates (*Acinetobacter* isolates DSM 1757) was sensitive to serum-mediated killing (Fig. 6); however, this sensitivity was abrogated after biofilm growth, even after disruption of the cells from the biofilm (Fig. 7). This latter finding suggests a biofilm-mediated change in gene/protein expression that affects serum sensitivity.

It is generally understood that laboratory serial passage of clinical isolates often results in isolate evolution that leads to changes in virulence and metabolic pathways; the change in selective pressures is believed to be responsible for this phenomenon (81). Based on this, we were surprised by how closely the common lab strains (PAO1 and AB5075) and ATCC reference strains (DSM 1718 and DSM 1719) clustered with the clinical isolates (Fig. 1; Fig. S3); laboratory strains that were isolated and serially passaged decades ago grouped closely with clinical isolates that were collected within the last several years. The *A. baumannii* strain ATCC 19606 (DSM 1719) was first isolated in 1948 (82) but grouped most closely to AB5075 on the phylogenetic tree (Fig. 1; Fig. S3); AB5075 is a Tibia/osteomyelitis isolate that was originally collected in 2008 (82) Fig. 1 and has become popular as a clinically relevant and virulent strain (77, 83). Likewise, *P. aeruginosa* ATCC 27853 (DSM 1718) grouped with PAO1, first isolated in 1954 (84), and clustered closely with the clinical isolates DSM 1745 through DSM 1748. Overall, these findings suggest that at a whole genomic level, these reference and laboratory strains remain representative of a subset of the circulating isolates of both species.

Multidrug resistance is defined as resistance to three or more categories of antimicrobials (51). While changes in antibiotic resistance in isolates from the same patient seemed to be driven by antibiotic treatment regimes, we were surprised by the apparent lack of multidrug resistance that was observed in the clinical isolates; only two of the 24 tested isolates were identified to fall into this category, and both of these were *P. aeruginosa*. Moreover, two of the *Acinetobacter* isolates and three of the *P. aeruginosa* isolates were sensitive to all of the tested antibiotics. Thus, although both *A. baumannii* and *P. aeruginosa* can gain antibiotic resistance via the acquisition of resistance genes from other organisms (16, 85) and can develop antibiotic resistance due to mutations (86, 87), clearly not all circulating clinical isolates of these species display antibiotic resistance profiles that may inhibit successful treatment.

Although we observed temporal and magnitude differences in biofilm formation, all tested isolates formed robust biofilms (Fig. 3 to 5). However, there were differences in how particular isolates formed biofilms in the 96-well plate as compared to the peg lid format (Fig. 3 and 4 versus Fig. 5); isolates that developed robust biofilm in one format often appeared to show a less robust biofilm in the other format. This may be driven by differences in plate material/surfaces between the two formats. Gram-negative bacteria typically have a preference for and accumulate on negatively charged, hydrophilic surfaces (88). The utilized 96-well plates are negatively charged, while the surface of the peg lid is hydrophobic, chargeless polystyrene. Thus, these differences in the surfaces may account for the biofilm formation profiles seen between the two assays. The results help to reinforce that biofilm formation can be affected by the chosen assay and material on which the biofilm is being formed.

*P. aeruginosa* sensitivity to serum-mediated killing is known to vary by strain as well as the type of infection the strain was isolated from (52, 89). Even among *P. aeruginosa* strains isolated from the bloodstream, only 34% of strains were sensitive to serum (89). However, we found that all of the liquid-grown *P. aeruginosa* isolates that we tested were resistant to serum-mediated killing (Fig. S2). Thus, these results suggest that *P. aeruginosa* wound isolates likely have a propensity for serum resistance. Analysis of a larger, diverse collection of *P. aeruginosa* wound isolates obtained from various locations would be needed to determine if our observed rates of serum resistance are similarly high in those isolates. For the *Acinetobacter* isolates, the DSM 1719 reference strain and the DSM 1757 clinical isolate were both sensitive to serum after growth in liquid culture (Fig. 6). However, growth of these strains in a biofilm abrogated this serum sensitivity (Fig. 7). Notably, this was true even though these cells were disrupted from the biofilm before being exposed to serum. Thus, growth within the biofilm condition likely results in changes in gene/protein expression that are responsible for the observed protection from serum-mediated killing. These results suggest that within an *in vivo* setting, cells contained within the biofilm matrix may display reduced sensitivity to serum-mediated killing. Moreover, individual cells released from the biofilm matrix due to natural or other means may also experience a period of serum resistance that could potentially help them survive and seed other areas of the body. Thus, further mechanistic work into this area may help to explain infection spread, even within the context of the natural defenses of the human body.

Finally, there are limitations to our study. The small number of patients and isolates limits our ability to make broader conclusions; a larger number of patients and isolates obtained over a larger timeframe could help to provide better insight into genotypic and phenotypic characteristics of wound isolates. It should also be noted that ANI defines similarity based on comparison of nucleotide sequences of shared genes but does not take into account the presence or absence of genes (90); thus, the completed analysis does not take into account the pan genome and may affect the overall phylogenetic relationships of the analyzed isolates. Additionally, the studies described here do not provide molecular insight into genetic features associated with any of the studied phenotypes. Future studies, like examining gene expression within the biofilm as compared to liquid culture, could help to identify genes that are crucial to observed phenotypes.

Regardless of these limitations, our basic characterization of circulating traumatic wound isolates of *P. aeruginosa* and *Acinetobacter* has provided additional insight into the genomes and phenotypic characteristics of these isolates as well as revealed areas for future studies to define components associated with the observed phenotypes.

## Data Availability

The data for all sequenced strains are deposited to NCBI (Bioproject PRJNA933552), and accession numbers for individual sequences are provided in Table 2.

## SUPPLEMENTAL MATERIAL

Supplemental figures spectrum.02644-25-s0001.pdf Figures S1 to S3.

Supplemental tables spectrum.02644-25-s0002.xlsx Tables S1 to S3.

ASM does not own the copyrights to Supplemental Material that may be linked to, or accessed through, an article. The authors have granted ASM a non-exclusive, world-wide license to publish the Supplemental Material files. Please contact the corresponding author directly for reuse.

## References

[1] Nussbaum SR, Carter MJ, Fife CE, DaVanzo J, Haught R, Nusgart M, Cartwright D. 2018. An economic evaluation of the impact, cost, and medicare policy implications of chronic nonhealing wounds. Value Health 21:27–32. doi:10.1016/j.jval.2017.07.007 29304937

[2] Tribble DR, Murray CK, Lloyd BA, Ganesan A, Mende K, Blyth DM, Petfield JL, McDonald J. 2019. After the battlefield: infectious complications among wounded warriors in the trauma infectious disease outcomes study. Mil Med 184:18–25. doi:10.1093/milmed/usz027 31778199 PMC6886670

[3] Sen CK, Gordillo GM, Roy S, Kirsner R, Lambert L, Hunt TK, Gottrup F, Gurtner GC, Longaker MT. 2009. Human skin wounds: a major and snowballing threat to public health and the economy. Wound Repair Regen 17:763–771. doi:10.1111/j.1524-475X.2009.00543.x 19903300 PMC2810192

[4] Tong MJ. 1972. Septic complications of war wounds. JAMA 219:1044–1047. 4621762

[5] Valentine KP, Viacheslav KM. 2017. Bacterial flora of combat wounds from eastern Ukraine and time-specified changes of bacterial recovery during treatment in Ukrainian military hospital. BMC Res Notes 10:152. doi:10.1186/s13104-017-2481-4 28388920 PMC5384141

[6] Sahli ZT, Bizri AR, Abu-Sittah GS. 2016. Microbiology and risk factors associated with war-related wound infections in the Middle East. Epidemiol Infect 144:2848–2857. doi:10.1017/S0950268816000431 26931769 PMC9150393

[7] Dow G, Browne A, Sibbald RG. 1999. Infection in chronic wounds: controversies in diagnosis and treatment. Ostomy Wound Manage 45:23–27. 10655866

[8] Peleg AY, Seifert H, Paterson DL. 2008. *Acinetobacter baumannii*: emergence of a successful pathogen. Clin Microbiol Rev 21:538–582. doi:10.1128/CMR.00058-07 18625687 PMC2493088

[9] Nathwani D, Raman G, Sulham K, Gavaghan M, Menon V. 2014. Clinical and economic consequences of hospital-acquired resistant and multidrug-resistant *Pseudomonas aeruginosa* infections: a systematic review and meta-analysis. Antimicrob Resist Infect Control 3:32. doi:10.1186/2047-2994-3-32 25371812 PMC4219028

[10] Shi Q, Huang C, Xiao T, Wu Z, Xiao Y. 2019. A retrospective analysis of *Pseudomonas aeruginosa* bloodstream infections: prevalence, risk factors, and outcome in carbapenem-susceptible and -non-susceptible infections. Antimicrob Resist Infect Control 8:68. doi:10.1186/s13756-019-0520-8 31057792 PMC6485151

[11] Gedefie A, Demsis W, Ashagrie M, Kassa Y, Tesfaye M, Tilahun M, Bisetegn H, Sahle Z. 2021. *Acinetobacter baumannii* Biofilm Formation and Its Role in Disease Pathogenesis: A Review. Infect Drug Resist 14:3711–3719. doi:10.2147/IDR.S332051 34531666 PMC8439624

[12] Gellatly SL, Hancock REW. 2013. *Pseudomonas aeruginosa*: new insights into pathogenesis and host defenses. Pathog Dis 67:159–173. doi:10.1111/2049-632X.12033 23620179

[13] Gale MJ, Maritato MS, Chen YL, Abdulateef SS, Ruiz JE. 2015. *Pseudomonas aeruginosa* causing inflammatory mass of the nasopharynx in an immunocompromised HIV infected patient: a mimic of malignancy. IDCases 2:40–43. doi:10.1016/j.idcr.2015.01.004 26793451 PMC4672612

[14] Ayoub Moubareck C, Hammoudi Halat D. 2020. Insights into *Acinetobacter baumannii*: a review of microbiological, virulence, and resistance traits in a threatening nosocomial pathogen. Antibiotics (Basel) 9:119. doi:10.3390/antibiotics9030119 32178356 PMC7148516

[15] Davis KA, Moran KA, McAllister CK, Gray PJ. 2005. Multidrug-resistan*t Acinetobacter* extremity infections in soldiers. Emerg Infect Dis 11:1218–1224. doi:10.3201/1108.050103 16102310 PMC3320488

[16] Pang Z, Raudonis R, Glick BR, Lin TJ, Cheng Z. 2019. Antibiotic resistance in *Pseudomonas aeruginosa*: mechanisms and alternative therapeutic strategies. Biotechnol Adv 37:177–192. doi:10.1016/j.biotechadv.2018.11.013 30500353

[17] Kang CI, Kim SH, Kim HB, Park SW, Choe YJ, Oh MD, Kim EC, Choe KW. 2003. *Pseudomonas aeruginosa* bacteremia: risk factors for mortality and influence of delayed receipt of effective antimicrobial therapy on clinical outcome. Clin Infect Dis 37:745–751. doi:10.1086/377200 12955633

[18] Clark NM, Zhanel GG, Lynch JP. 2016. Emergence of antimicrobial resistance among Acinetobacter species: a global threat. Curr Opin Crit Care 22:491–499. doi:10.1097/MCC.0000000000000337 27552304

[19] Thi MTT, Wibowo D, Rehm BHA. 2020*.* *Pseudomonas aeruginosa* biofilms. IJMS 21:8671. doi:10.3390/ijms21228671 33212950 PMC7698413

[20] Solomon SL, Oliver KB. 2014. Antibiotic resistance threats in the United States: stepping back from the brink. Am Fam Physician 89:938–941. 25162160

[21] Flemming HC, Wingender J. 2010. The biofilm matrix. Nat Rev Microbiol 8:623–633. doi:10.1038/nrmicro2415 20676145

[22] Costerton JW, Lewandowski Z, Caldwell DE, Korber DR, Lappin-Scott HM. 1995. Microbial biofilms. Annu Rev Microbiol 49:711–745. doi:10.1146/annurev.mi.49.100195.003431 8561477

[23] Stewart PS, Franklin MJ. 2008. Physiological heterogeneity in biofilms. Nat Rev Microbiol 6:199–210. doi:10.1038/nrmicro1838 18264116

[24] Hall-Stoodley L, Costerton JW, Stoodley P. 2004. Bacterial biofilms: from the natural environment to infectious diseases. Nat Rev Microbiol 2:95–108. doi:10.1038/nrmicro821 15040259

[25] Stewart PS, Costerton JW. 2001. Antibiotic resistance of bacteria in biofilms. Lancet 358:135–138. doi:10.1016/s0140-6736(01)05321-1 11463434

[26] Leid JG, Shirtliff ME, Costerton JW, Stoodley P. 2002. Human leukocytes adhere to, penetrate, and respond to S*taphylococcus aureus* biofilms. Infect Immun 70:6339–6345. doi:10.1128/IAI.70.11.6339-6345.2002 12379713 PMC130380

[27] Domenech M, Ramos-Sevillano E, García E, Moscoso M, Yuste J. 2013. Biofilm formation avoids complement immunity and phagocytosis of *Streptococcus pneumoni*ae. Infect Immun 81:2606–2615. doi:10.1128/IAI.00491-13 23649097 PMC3697597

[28] Vuong C, Kocianova S, Voyich JM, Yao Y, Fischer ER, DeLeo FR, Otto M. 2004. A crucial role for exopolysaccharide modification in bacterial biofilm formation, immune evasion, and virulence. J Biol Chem 279:54881–54886. doi:10.1074/jbc.M411374200 15501828

[29] Mann EE, Wozniak DJ. 2012. *Pseudomonas* biofilm matrix composition and niche biology. FEMS Microbiol Rev 36:893–916. doi:10.1111/j.1574-6976.2011.00322.x 22212072 PMC4409827

[30] Costerton JW, Stewart PS, Greenberg EP. 1999. Bacterial biofilms: a common cause of persistent infections. Science 284:1318–1322. doi:10.1126/science.284.5418.1318 10334980

[31] James GA, Swogger E, Wolcott R, Pulcini E deLancey, Secor P, Sestrich J, Costerton JW, Stewart PS. 2008. Biofilms in chronic wounds. Wound Repair Regeneration 16:37–44. doi:10.1111/j.1524-475X.2007.00321.x 18086294

[32] Jung L, Kiwanuka J, Mbabazi L, Nakate V, Musaazi J, Nabajja H, Kajumbula H, Lübbert C, Mwaka E, Nsibirwa S, von Braun A. 2023. A case for routine microbial diagnostics: results from antimicrobial susceptibility testing in post-traumatic wound infections at a Ugandan tertiary care hospital. PLoS Glob Public Health 3:e0001880. doi:10.1371/journal.pgph.0001880 37582103 PMC10427013

[33] Bartow‐McKenney C, Hannigan GD, Horwinski J, Hesketh P, Horan AD, Mehta S, Grice EA. 2018. The microbiota of traumatic, open fracture wounds is associated with mechanism of injury. Wound Repair Regeneration 26:127–135. doi:10.1111/wrr.12642 29802752 PMC6202213

[34] Culyba MJ, Van Tyne D. 2021. Bacterial evolution during human infection: adapt and live or adapt and die. PLoS Pathog 17:e1009872. doi:10.1371/journal.ppat.1009872 34499699 PMC8428787

[35] Lisboa FA, Dente CJ, Schobel SA, Khatri V, Potter BK, Kirk AD, Elster EA. 2019. Utilizing precision medicine to estimate timing for surgical closure of traumatic extremity wounds. Ann Surg 270:535–543. doi:10.1097/SLA.0000000000003470 31348045

[36] Lisboa FA, Bradley MJ, Hueman MT, Schobel SA, Gaucher BJ, Styrmisdottir EL, Potter BK, Forsberg JA, Elster EA. 2017. Nonsteroidal anti-inflammatory drugs may affect cytokine response and benefit healing of combat-related extremity wounds. Surgery 161:1164–1173. doi:10.1016/j.surg.2016.10.011 27919449

[37] Sheppard FR, Keiser P, Craft DW, Gage F, Robson M, Brown TS, Petersen K, Sincock S, Kasper M, Hawksworth J, Tadaki D, Davis TA, Stojadinovic A, Elster E. 2010. The majority of US combat casualty soft-tissue wounds are not infected or colonized upon arrival or during treatment at a continental US military medical facility. Am J Surg 200:489–495. doi:10.1016/j.amjsurg.2010.03.001 20887842

[38] Brown TS, Hawksworth JS, Sheppard FR, Tadaki DK, Elster E. 2011. Inflammatory response is associated with critical colonization in combat wounds. Surg Infect (Larchmt) 12:351–357. doi:10.1089/sur.2010.110 21936666

[39] Humphries R, Bobenchik AM, Hindler JA, Schuetz AN. 2021. Overview of changes to the clinical and laboratory standards institute *Performance Standards for Antimicrobial Susceptibility Testing,* M100, 31st edition. J Clin Microbiol 59:e0021321. doi:10.1128/JCM.00213-21 34550809 PMC8601225

[40] Evans DG, Evans DJ, Tjoa W. 1977. Hemagglutination of human group A erythrocytes by enterotoxigenic *Escherichia coli* isolated from adults with diarrhea: correlation with colonization factor. Infect Immun 18:330–337. doi:10.1128/iai.18.2.330-337.1977 336541 PMC421235

[41] Liu B, Zheng D, Jin Q, Chen L, Yang J. 2019. VFDB 2019: a comparative pathogenomic platform with an interactive web interface. Nucleic Acids Res 47:D687–D692. doi:10.1093/nar/gky1080 30395255 PMC6324032

[42] Bankevich A, Nurk S, Antipov D, Gurevich AA, Dvorkin M, Kulikov AS, Lesin VM, Nikolenko SI, Pham S, Prjibelski AD, Pyshkin AV, Sirotkin AV, Vyahhi N, Tesler G, Alekseyev MA, Pevzner PA. 2012. SPAdes: a new genome assembly algorithm and its applications to single-cell sequencing. J Comput Biol 19:455–477. doi:10.1089/cmb.2012.0021 22506599 PMC3342519

[43] Gurevich A, Saveliev V, Vyahhi N, Tesler G. 2013. QUAST: quality assessment tool for genome assemblies. Bioinformatics 29:1072–1075. doi:10.1093/bioinformatics/btt086 23422339 PMC3624806

[44] Parks DH, Imelfort M, Skennerton CT, Hugenholtz P, Tyson GW. 2015. CheckM: assessing the quality of microbial genomes recovered from isolates, single cells, and metagenomes. Genome Res 25:1043–1055. doi:10.1101/gr.186072.114 25977477 PMC4484387

[45] Wu Y-W, Simmons BA, Singer SW. 2016. MaxBin 2.0: an automated binning algorithm to recover genomes from multiple metagenomic datasets. Bioinformatics 32:605–607. doi:10.1093/bioinformatics/btv638 26515820

[46] Alcock BP, Huynh W, Chalil R, Smith KW, Raphenya AR, Wlodarski MA, Edalatmand A, Petkau A, Syed SA, Tsang KK, et al.. 2023. CARD 2023: expanded curation, support for machine learning, and resistome prediction at the comprehensive antibiotic resistance database. Nucleic Acids Res 51:D690–D699. doi:10.1093/nar/gkac920 36263822 PMC9825576

[47] Emms DM, Kelly S. 2019. OrthoFinder: phylogenetic orthology inference for comparative genomics. Genome Biol 20:238. doi:10.1186/s13059-019-1832-y 31727128 PMC6857279

[48] Olson RD, Assaf R, Brettin T, Conrad N, Cucinell C, Davis JJ, Dempsey DM, Dickerman A, Dietrich EM, Kenyon RW, et al.. 2023. Introducing the bacterial and viral bioinformatics resource center (BV-BRC): a resource combining PATRIC, IRD and ViPR. Nucleic Acids Res 51:D678–D689. doi:10.1093/nar/gkac1003 36350631 PMC9825582

[49] Merritt JH, Kadouri DE, O’Toole GA. 2006. Growing and analyzing static biofilms. CP Microbiology 00:1. doi:10.1002/9780471729259.mc01b01s00 PMC4568995 18770545

[50] Wu H, Jerse AE. 2006. Alpha-2,3-sialyltransferase enhances *Neisseria gonorrhoeae* survival during experimental murine genital tract infection. Infect Immun 74:4094–4103. doi:10.1128/IAI.00433-06 16790783 PMC1489707

[51] Magiorakos AP, Srinivasan A, Carey RB, Carmeli Y, Falagas ME, Giske CG, Harbarth S, Hindler JF, Kahlmeter G, Olsson-Liljequist B, Paterson DL, Rice LB, Stelling J, Struelens MJ, Vatopoulos A, Weber JT, Monnet DL. 2012. Multidrug-resistant, extensively drug-resistant and pandrug-resistant bacteria: an international expert proposal for interim standard definitions for acquired resistance. Clin Microbiol Infect 18:268–281. doi:10.1111/j.1469-0691.2011.03570.x 21793988

[52] Vitkauskiene A, Scheuss S, Sakalauskas R, Dudzevicius V, Sahly H. 2005. *Pseudomonas aeruginosa* strains from nosocomial pneumonia are more serum resistant than *P. aeruginosa* strains from noninfectious respiratory colonization processes. Infection 33:356–361. doi:10.1007/s15010-005-5044-x 16258867

[53] Wang D, Wang L, Liu Q, Zhao Y. 2025. Virulence factors in biofilm formation and therapeutic strategies for *Staphylococcus aureus*: a review. Animals and Zoonoses 1:188–202. doi:10.1016/j.azn.2024.11.003

[54] Boulesnam SL, Hamaidi-Chergui F, Benamara M, Azrou S. 2023. Phenotypical comparison between environmental and clinical *Acinetobacter baumannii* strains isolated from an intensive care unit. Malays J Med Sci 30:85–93. doi:10.21315/mjms2023.30.4.8 37655144 PMC10467598

[55] Villalón P, Ortega M, Sáez-Nieto JA, Carrasco G, Medina-Pascual MJ, Garrido N, Valdezate S. 2019. Dynamics of a sporadic nosocomial *Acinetobacter calcoaceticus - Acinetobacter baumannii* complex population. Front Microbiol 10:593. doi:10.3389/fmicb.2019.00593 30967856 PMC6440288

[56] Nemec A, Krizova L, Maixnerova M, Sedo O, Brisse S, Higgins PG. 2015. *Acinetobacter seifertii* sp. nov., a member of the A*cinetobacter calcoaceticus-Acinetobacter baumannii* complex isolated from human clinical specimens. Int J Syst Evol Microbiol 65:934–942. doi:10.1099/ijs.0.000043 25563912

[57] Cosgaya C, Marí-Almirall M, Van Assche A, Fernández-Orth D, Mosqueda N, Telli M, Huys G, Higgins PG, Seifert H, Lievens B, Roca I, Vila J. 2016. *Acinetobacter dijkshoorniae* sp. nov., a member of the *Acinetobacter calcoaceticus-Acinetobacter baumannii* complex mainly recovered from clinical samples in different countries. Int J Syst Evol Microbiol 66:4105–4111. doi:10.1099/ijsem.0.001318 27432448

[58] Wolf S, Barth-Jakschic E, Birkle K, Bader B, Marschal M, Liese J, Peter S, Oberhettinger P. 2021. *Acinetobacter geminorum* sp. nov., isolated from human throat swabs. Int J Syst Evol Microbiol 71:005018. doi:10.1099/ijsem.0.005018 34633923 PMC8604166

[59] Kang YS, Jung J, Jeon CO, Park W. 2011. *Acinetobacter oleivorans* sp. nov. is capable of adhering to and growing on diesel-oil. J Microbiol 49:29–34. doi:10.1007/s12275-011-0315-y 21369976

[60] Sheck E, Romanov A, Shapovalova V, Shaidullina E, Martinovich A, Ivanchik N, Mikotina A, Skleenova E, Oloviannikov V, Azizov I, Vityazeva V, Lavrinenko A, Kozlov R, Edelstein M. 2023. *Acinetobacter* non-*baumannii* species: occurrence in infections in hospitalized patients, identification, and antibiotic resistance. Antibiotics (Basel) 12:1301. doi:10.3390/antibiotics12081301 37627721 PMC10451542

[61] Gerner-Smidt P. 1992. Ribotyping of the *Acinetobacter calcoaceticus*-*Acinetobacter baumannii* complex. J Clin Microbiol 30:2680–2685. doi:10.1128/jcm.30.10.2680-2685.1992 1383266 PMC270498

[62] Gerner-Smidt P, Tjernberg I, Ursing J. 1991. Reliability of phenotypic tests for identification of *Acinetobacter* species. J Clin Microbiol 29:277–282. doi:10.1128/jcm.29.2.277-282.1991 2007635 PMC269753

[63] Bernards AT, van der Toorn J, van Boven CP, Dijkshoorn L. 1996. Evaluation of the ability of a commercial system to identify *Acinetobacter* genomic species. Eur J Clin Microbiol Infect Dis 15:303–308. doi:10.1007/BF01695662 8781881

[64] Horrevorts A, Bergman K, Kollée L, Breuker I, Tjernberg I, Dijkshoorn L. 1995. Clinical and epidemiological investigations of *Acinetobacter genomospecies* 3 in a neonatal intensive care unit. J Clin Microbiol 33:1567–1572. doi:10.1128/jcm.33.6.1567-1572.1995 7650188 PMC228217

[65] Vijayakumar S, Biswas I, Veeraraghavan B. 2019. Accurate identification of clinically important *Acinetobacter* spp.: an update. Future Sci OA 5:FSO395. doi:10.2144/fsoa-2018-0127 31285840 PMC6609899

[66] Chuang YC, Sheng WH, Li SY, Lin YC, Wang JT, Chen YC, Chang SC. 2011. Influence of genospecies of *Acinetobacter baumannii* complex on clinical outcomes of patients with *Acinetobacter* bacteremia. Clin Infect Dis 52:352–360. doi:10.1093/cid/ciq154 21193494

[67] Bartual SG, Seifert H, Hippler C, Luzon MAD, Wisplinghoff H, Rodríguez-Valera F. 2005. Development of a multilocus sequence typing scheme for characterization of clinical isolates of *Acinetobacter baumannii*. J Clin Microbiol 43:4382–4390. doi:10.1128/JCM.43.9.4382-4390.2005 16145081 PMC1234098

[68] Chen L, Yang J, Yu J, Yao Z, Sun L, Shen Y, Jin Q. 2004. VFDB: a reference database for bacterial virulence factors. Nucleic Acids Res 33:D325–D328. doi:10.1093/nar/gki008 PMC539962 15608208

[69] Sobel ML, Hocquet D, Cao L, Plesiat P, Poole K. 2005. Mutations in PA3574 (nalD) lead to increased MexAB-OprM expression and multidrug resistance in laboratory and clinical isolates of *Pseudomonas aeruginosa*. Antimicrob Agents Chemother 49:1782–1786. doi:10.1128/AAC.49.5.1782-1786.2005 15855496 PMC1087681

[70] Pérez-Varela M, Corral J, Aranda J, Barbé J. 2018. Functional characterization of AbaQ, a novel efflux pump mediating quinolone resistance in *Acinetobacter baumannii*. Antimicrob Agents Chemother 62:e00906-18. doi:10.1128/AAC.00906-18 29941648 PMC6125561

[71] Nie L, Lv Y, Yuan M, Hu X, Nie T, Yang X, Li G, Pang J, Zhang J, Li C, Wang X, You X. 2014. Genetic basis of high level aminoglycoside resistance in *Acinetobacter baumannii* from Beijing, China. Acta Pharm Sin B 4:295–300. doi:10.1016/j.apsb.2014.06.004 26579398 PMC4629078

[72] Mack AR, Hujer AM, Mojica MF, Taracila MA, Feldgarden M, Haft DH, Klimke W, Prasad AB, Bonomo RA. 2025. β-Lactamase diversity in *Acinetobacter baumannii*. Antimicrob Agents Chemother:e0078424. doi:10.1128/aac.00784-24 39927782 PMC11881555

[73] Bhattacharya M, Toth M, Antunes NT, Smith CA, Vakulenko SB. 2014. Structure of the extended-spectrum class C β-lactamase ADC-1 from *Acinetobacter baumannii*. Acta Crystallogr D Biol Crystallogr 70:760–771. doi:10.1107/S1399004713033014 24598745 PMC3949520

[74] Hahn HP. 1997. The type-4 pilus is the major virulence-associated adhesin of *Pseudomonas aeruginosa*--a review. Gene 192:99–108. doi:10.1016/s0378-1119(97)00116-9 9224879

[75] Ren G, Wang Z, Li Y, Hu X, Wang X. 2016. Effects of lipopolysaccharide core sugar deficiency on colanic acid biosynthesis in *Escherichia coli*. J Bacteriol 198:1576–1584. doi:10.1128/JB.00094-16 27002133 PMC4959291

[76] Sakhtah H, Price-Whelan A, Dietrich L. 2013. Regulation of phenazine biosynthesis, p 19–42. *In* Microbial phenazines: biosynthesis, agriculture and health

[77] Williams CL, Neu HM, Alamneh YA, Reddinger RM, Jacobs AC, Singh S, Abu-Taleb R, Michel SLJ, Zurawski DV, Merrell DS. 2020. Characterization of *Acinetobacter baumannii* copper resistance reveals a role in virulence. Front Microbiol 11:16. doi:10.3389/fmicb.2020.00016 32117089 PMC7015863

[78] Tuon FF, Dantas LR, Suss PH, Tasca Ribeiro VS. 2022. Pathogenesis of the *Pseudomonas aeruginosa* biofilm: a review. Pathogens 11:300. doi:10.3390/pathogens11030300 35335624 PMC8950561

[79] Amala Reena AA, Subramaniyan A, Kanungo R. 2017. Biofilm formation as a virulence factor of *Acinetobacter baumannii*: an emerging pathogen in critical care units. J Curr Res Sci Med 3:74. doi:10.4103/jcrsm.jcrsm_66_17

[80] Masila EM, Too E. 2024. The interconnection between virulence factors, biofilm formation, and horizontal gene transfer in enterococcus: a review. *In* Téllez-Isaías G, Graham D, El-Ashram S, Gray L (ed), Enterococcus - unveiling the emergence of a potent pathogen. IntechOpen, Rijeka.

[81] Fux CA, Shirtliff M, Stoodley P, Costerton JW. 2005. Can laboratory reference strains mirror “real-world” pathogenesis? Trends Microbiol 13:58–63. doi:10.1016/j.tim.2004.11.001 15680764

[82] Hugh R, Reese R. 1967. Designation of the type strain for bacterium anitratum Schaub and Hauber 1948. Int J Syst Bacteriol 17:245–254. doi:10.1099/00207713-17-3-245

[83] Jacobs AC, Thompson MG, Black CC, Kessler JL, Clark LP, McQueary CN, Gancz HY, Corey BW, Moon JK, Si Y, et al.. 2014. AB5075, a highly virulent isolate of *Acinetobacter baumanni*i, as a model strain for the evaluation of pathogenesis and antimicrobial treatments. mBio 5:e01076-14. doi:10.1128/mBio.01076-14 24865555 PMC4045072

[84] Chandler CE, Horspool AM, Hill PJ, Wozniak DJ, Schertzer JW, Rasko DA, Ernst RK. 2019. Genomic and phenotypic diversity among ten laboratory isolates of *Pseudomonas aeruginosa* PAO1. J Bacteriol 201:e00595-18. doi:10.1128/JB.00595-18 30530517 PMC6379574

[85] Vázquez-López R, Solano-Gálvez SG, Juárez Vignon-Whaley JJ, Abello Vaamonde JA, Padró Alonzo LA, Rivera Reséndiz A, Muleiro Álvarez M, Vega López EN, Franyuti-Kelly G, Álvarez-Hernández DA, Moncaleano Guzmán V, Juárez Bañuelos JE, Marcos Felix J, González Barrios JA, Barrientos Fortes T. 2020. *Acinetobacter baumannii* resistance: a real challenge for clinicians. Antibiotics (Basel) 9:205. doi:10.3390/antibiotics9040205 32340386 PMC7235888

[86] Breidenstein EBM, de la Fuente-Núñez C, Hancock REW. 2011. *Pseudomonas aeruginosa*: all roads lead to resistance. Trends Microbiol 19:419–426. doi:10.1016/j.tim.2011.04.005 21664819

[87] Kyriakidis I, Vasileiou E, Pana ZD, Tragiannidis A. 2021. *Acinetobacter baumannii* Antibiotic Resistance Mechanisms. Pathogens 10:373. doi:10.3390/pathogens10030373 33808905 PMC8003822

[88] Gottenbos B, Grijpma DW, van der Mei HC, Feijen J, Busscher HJ. 2001. Antimicrobial effects of positively charged surfaces on adhering Gram-positive and Gram-negative bacteria. J Antimicrob Chemother 48:7–13. doi:10.1093/jac/48.1.7 11418507

[89] Hickson SM, Hoehensteiger JK, Mayer-Coverdale J, Torres VVL, Feng W, Monteith JN, Henderson IR, McCarthy KL, Wells TJ. 2024. Antibody-mediated serum resistance protects *Pseudomonas aeruginosa* during bloodstream infections. J Infect Dis 230:e221–e229. doi:10.1093/infdis/jiad457 38235716 PMC11326846

[90] Rodriguez-R LM, Conrad RE, Viver T, Feistel DJ, Lindner BG, Venter SN, Orellana LH, Amann R, Rossello-Mora R, Konstantinidis KT. 2024. An ANI gap within bacterial species that advances the definitions of intra-species units. mBio 15:e0269623. doi:10.1128/mbio.02696-23 38085031 PMC10790751

