# Supplementary material for: Characterization of Pseudomonas aeruginosa and Acinetobacter calcoaceticus-baumannii complex traumatic wound isolates
Source: Microbiol Spectr. 2026 Jun 12;14(7):e02644-25. doi: 10.1128/spectrum.02644-25 (PMC13340109; doi:10.1128/spectrum.02644-25)
Supplement: Supplemental figures — Figures S1 to S3. [file spectrum.02644-25-s0001.pdf]

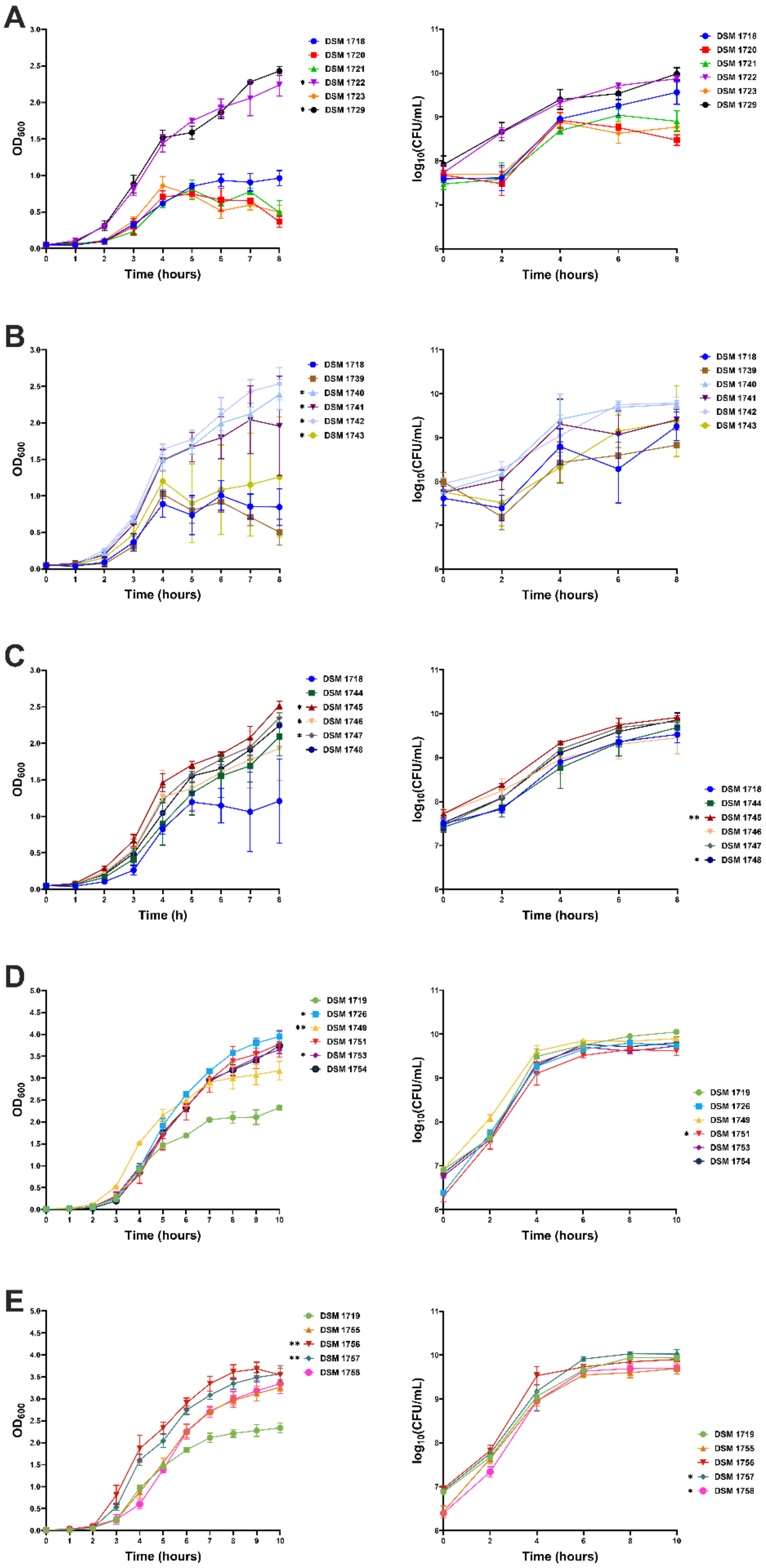
A
B
C
D
E
OD600
log10(CFU/mL)
Time (hours)
Time (h)
DSM 1718
DSM 1720
DSM 1721
DSM 1722
DSM 1723
DSM 1729
DSM 1739
DSM 1740
DSM 1741
DSM 1742
DSM 1743
DSM 1744
DSM 1745
DSM 1746
DSM 1747
DSM 1748
DSM 1719
DSM 1726
DSM 1749
DSM 1751
DSM 1753
DSM 1754
DSM 1755
DSM 1756
DSM 1757
DSM 1758

Supplemental Figure 1. Growth curves of indicated *P. aeruginosa* and *Acinetobacter* isolates**.** Isolates of *P. aeruginosa* (A-C) and *Acinetobacter* (D-E) were grown in liquid culture at 37°C shaking at 190 RPM. OD_{600} (left) were measured every hour and CFU (right) were enumerated by plating every 2 hours. CFU data were log-transformed for plotting and analysis. Data were obtained from three biologically independent replicates and the mean is plotted; error bars display standard error of the mean (OD_{600} data) or standard deviation (CFU data); LOD = limit of detection. Repeated measures one-way ANOVAs with Dunnett correction for multiple comparisons were performed; *$p<0.05$ and, **$p<0.01$ as compared to DSM 1718 for *P. aeruginosa* and DSM 1719 for *Acinetobacter.*

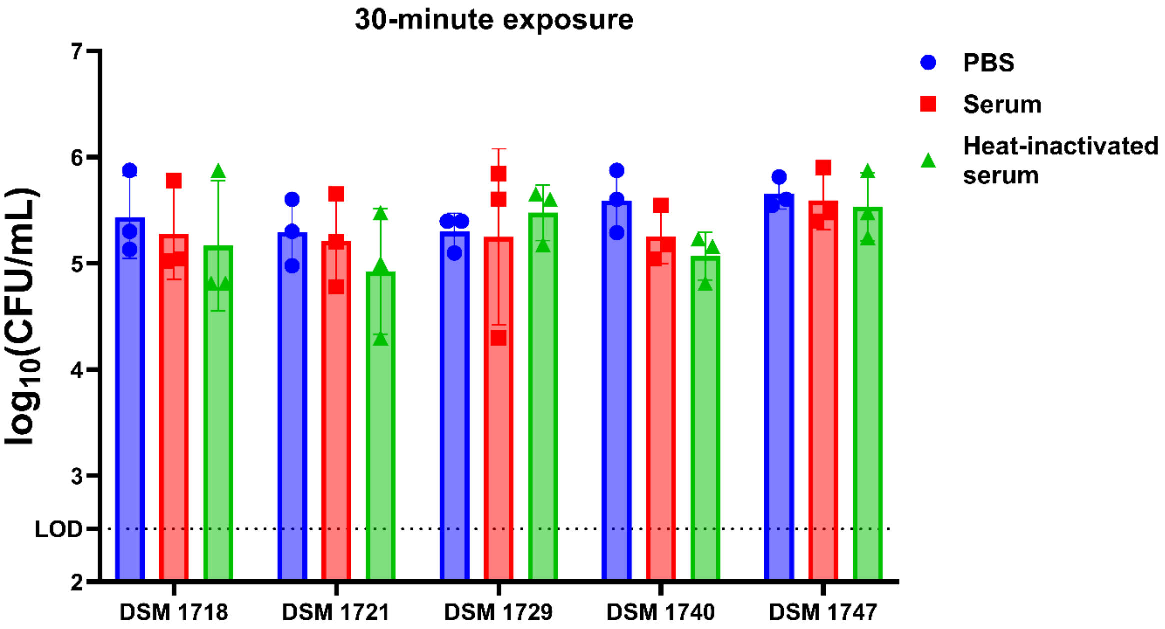

Supplemental Figure 2. Serum resistance of *P. aeruginosa* cells from liquid culture. Cultures of *P. aeruginosa* isolates were grown for 3 hours and OD adjusted to OD_{600} of 0.05. Next, 2.5 µL of this adjusted culture (~10^5 cells) were added to serum, PBS or heat-inactivated serum for 30 minutes. After 30 minutes the complement reaction of the serum was stopped via the addition of EDTA; the surviving CFU were enumerated and log-transformed for plotting and analysis. Data were obtained from three biologically independent replicates; individual data points and the mean is plotted; error bars display standard deviation; LOD = limit of detection. A two-way ANOVA with Dunnett correction for multiple comparisons was performed; no significant differences were

detected as compared to the PBS control.

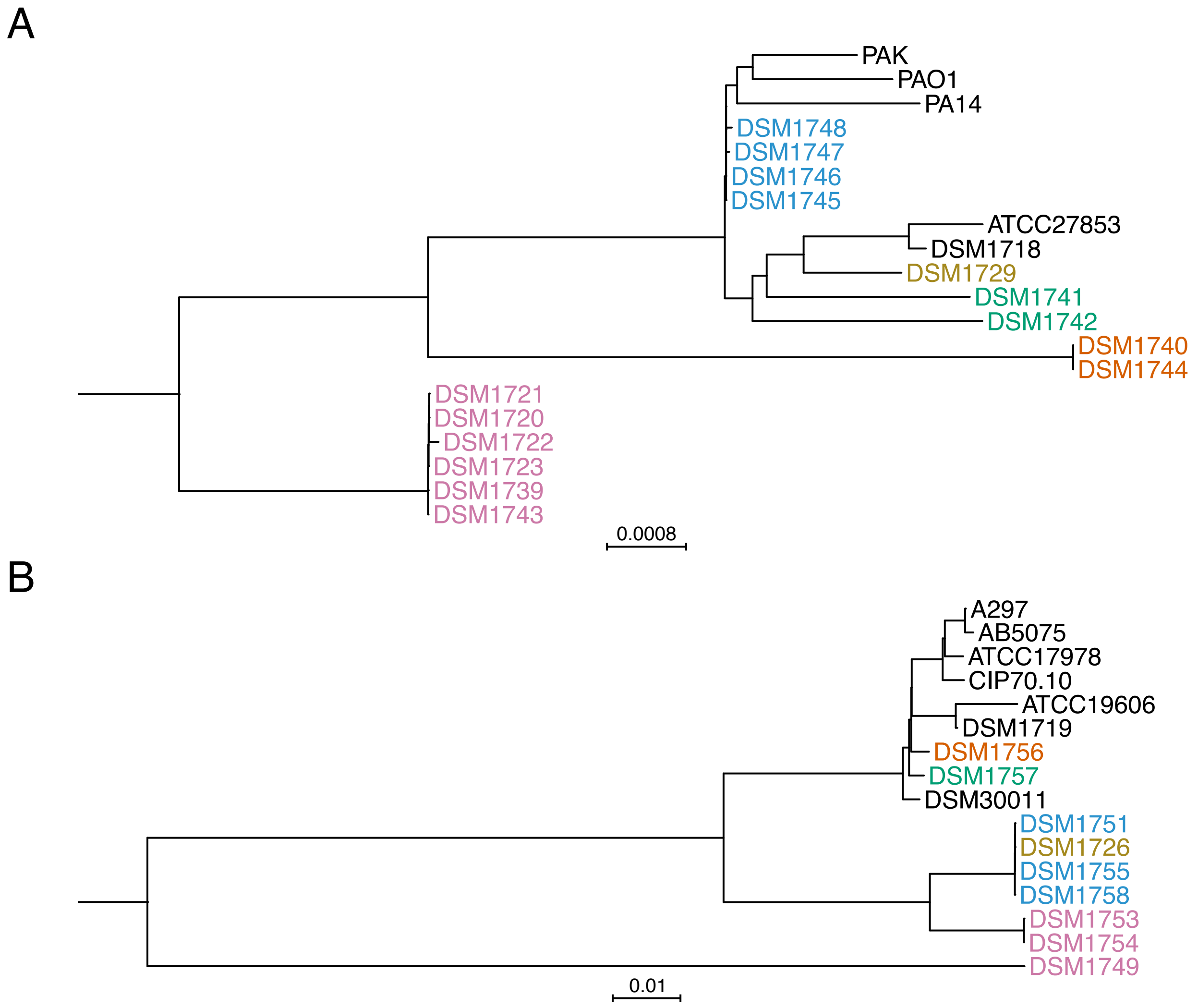

Supplemental Figure 3. Phylogenetic relationship of *P. aeruginosa* and *Acinetobacter* clinical isolates and expanded collection of reference strains. Species trees were constructed from 4,461 and 1,954 conserved, single-copy genes for *P. aeruginosa* (A) and *Acinetobacter* (B), respectively. Isolates obtained from the same patient are denoted by the use of the same color for the strain name designations. Reference strains are colored in black. Branch length and scale bar refer to the mean amino acid substitutions per site (*e.g.* branch length of 0.02 is equal to 1 amino acid change per 50 sites).
